# Cell death induction and protection by activation of ubiquitously expressed anion/cation channels. Part 3: the roles and properties of TRPM2 and TRPM7

**DOI:** 10.3389/fcell.2023.1246955

**Published:** 2023-09-29

**Authors:** Yasunobu Okada, Tomohiro Numata, Ravshan Z. Sabirov, Makiko Kashio, Peter G. Merzlyak, Kaori Sato-Numata

**Affiliations:** ^1^ National Institute for Physiological Sciences (NIPS), Okazaki, Japan; ^2^ Department of Integrative Physiology, Graduate School of Medicine, AkitaUniversity, Akita, Japan; ^3^ Department of Physiology, School of Medicine, Aichi Medical Uniersity, Nagakute, Japan; ^4^ Department of Physiology, Kyoto Prefectural University of Medicine, Kyoto, Japan; ^5^ Cardiovascular Research Institute, Yokohama City University, Yokohama, Japan; ^6^ Institute of Biophysics and Biochemistry, National University of Uzbekistan, Tashkent, Uzbekistan

**Keywords:** cell volume regulation, apoptosis, necrosis, pyroptosis, oxidative cell death, acidotoxic cell death, TRPM2, TRPM7

## Abstract

Cell volume regulation (CVR) is a prerequisite for animal cells to survive and fulfill their functions. CVR dysfunction is essentially involved in the induction of cell death. In fact, sustained normotonic cell swelling and shrinkage are associated with necrosis and apoptosis, and thus called the necrotic volume increase (NVI) and the apoptotic volume decrease (AVD), respectively. Since a number of ubiquitously expressed ion channels are involved in the CVR processes, these volume-regulatory ion channels are also implicated in the NVI and AVD events. In Part 1 and Part 2 of this series of review articles, we described the roles of swelling-activated anion channels called VSOR or VRAC and acid-activated anion channels called ASOR or PAC in CVR and cell death processes. Here, Part 3 focuses on therein roles of Ca^2+^-permeable non-selective TRPM2 and TRPM7 cation channels activated by stress. First, we summarize their phenotypic properties and molecular structure. Second, we describe their roles in CVR. Since cell death induction is tightly coupled to dysfunction of CVR, third, we focus on their participation in the induction of or protection against cell death under oxidative, acidotoxic, excitotoxic, and ischemic conditions. In this regard, we pay attention to the sensitivity of TRPM2 and TRPM7 to a variety of stress as well as to their capability to physicall and functionally interact with other volume-related channels and membrane enzymes. Also, we summarize a large number of reports hitherto published in which TRPM2 and TRPM7 channels are shown to be involved in cell death associated with a variety of diseases or disorders, in some cases as double-edged swords. Lastly, we attempt to describe how TRPM2 and TRPM7 are organized in the ionic mechanisms leading to cell death induction and protection.

## Introduction

Animal cells must regulate their cell volume even under physiological conditions with constant extracellular osmolarity. First, it is because the fluctuation of intracellular osmolarity is inevitably induced by cell activity *per se* which constantly requires osmolyte transport across the cell membrane and cell metabolism (anabolism and catabolism). Second, it is because cell volume changes are coupled to cell migration and cell proliferation connoting mitosis. After cell swelling and shrinkage, animal cells can shortly regulate their volume. The mechanisms of cell volume regulation are called the regulatory volume decrease (RVD) and the regulatory volume increase (RVI) that are attained by water movements driven by KCl efflux and NaCl influx, respectively (Hoffmann et al., 2009; Lang et al., 1998; Okada, 2004). In most types of mammalian cells, such volume-regulatory KCl and NaCl transports are principally accomplished by the activities of ubiquitously expressed anion and cation channels.

When the CVR mechanisms are persistently impaired, cells cannot survive. Sustained cell shrinkage and swelling are major hallmarks of the early, presumably earliest, events of necrotic and apoptotic cell death, and thus called the apoptotic volume decrease (AVD) (Maeno et al., 2000) and the necrotic volume increase (NVI) (Barros et al., 2001; Okada et al., 2001), respectively. Thus, these cell death processes are associated with dysregulation of a variety of ion transport mechanisms especially ion channels (Ritter et al., 2021). In the previous Part 1 article (Okada et al., 2021a) and Part 2 article (Okada et al., 2021b), we summarized the roles of the volume-sensitive outwardly rectifying anion channel (VSOR) (Okada, 1997), also called the volume-regulated anion channel (VRAC) (Nilius et al., 1997a), and those of the acid-sensitive outwardly rectifying anion channel (ASOR) (Wang et al., 2007), also called the proton-activated anion channel (PAC) (Yang et al., 2019), respectively, in CVR and cell death induction. To attain net KCl and NaCl transports, these anion channels should operate in parallel with K^+^ channels and Na^+^-permeable cation channels due to the electroneutrality constraint. In fact, altered activities of TRPM2 and TRPM7 cation channels are known to be implicated in CVR function/dysfunction and cell death induction. Here, we review the roles of TRPM2 and TRPM7 channels in the processes of CVR and cell death induction in this Part 3 article.

## Phenotypic properties and molecular structures of TRPM2 and TRPM7

### Chanzymes TRPM2 and TRPM7 as the members of sensor TRP channels

The Transient Receptor Potential (TRP) ion channel family consists of a large number (28 for human) of members, and is subdivided into 6 subfamilies in mammals: TRPC (“Canonical” or “Classical”), TRPM (“Melastatin”), TRPV (“Vanilloid”), TRPA (“Ankyrin”), TRPML (“MucoLipin”), and TRPP (PKD or “Polycystin”). TRP channels have a tetrameric subunit stoichiometry, with each subunit containing the cytoplasmic *N*- and *C*-terminal regions, six transmembrane domains (S1∼S6), and a pore-forming region between S5 and S6. TRP channels are non-selective cation-conductive membrane proteins and play a central role in physiological processes involving ionic signals. TRP channels are polymodal ion channels that have the role of integrating and transmitting a variety of environmental stimuli, including physical stimuli such as mechanical and thermal, and/or chemical stimuli such as pH and plant-derived compounds. These features serve as sensors to monitor the body’s extrinsic and intrinsic abnormalities while providing the basis for maintaining homeostasis that controls adaptive signals. Therefore, these are closely linked to health and disease and are attractive targets for drug discovery.

TRPM, the largest TRP subfamily, contains four melastatin domains, that are TRPM homology regions (MHR1-4), at the *N*-terminus and functions in a wide variety of cells throughout our body, including homeostasis-related cell proliferation, metabolism, cell death, and cancer (Dhakal and Lee, 2019; Fliniaux et al., 2018; Hantute-Ghesquier et al., 2018; Wong et al., 2019). As a main example, TRPM1: ON bipolar function of the retina (Koike et al., 2010), TRPM2: oxidative stress sensor function of cells and tissues (Naziroğlu, 2007), TRPM3: thermal sensor function (Vriens and Voets, 2019), TRPM4: regulator of cardiac conduction (Wang C. et al., 2018), TRPM5: taste sensor and blood glucose control capability (Vennekens et al., 2018), TRPM6: regulation of magnesium homeostasis (de Baaij et al., 2015), TRPM7: mechano-sensor function in cells (Numata et al., 2017a; Numata et al., 2017b) as well as synaptic and cognitive functions in the nervous system (Abumaria et al., 2019), and TRPM8: cold sensor function (Liu et al., 2020). Among these sensor TRPM channels, TRPM2, TRPM3, TRPM6, TRPM7, and TRPM8 are Ca^2+^-permeable cation channels activated by stress. In particular, TRPM2 and TRPM7 are unique ion channels possessing both ion channel and enzyme structures/activities and are called chanzymes

### Physiological roles of TRPM2 and TRPM7

The activities of TRPM2 and TRPM7 are involved in a variety of physiological functions, as shortly summarized below.

#### TRPM2

The TRPM2 channel was first cloned by Nagamine et al. (Nagamine et al., 1998) and is a homo-tetrameric, non-voltage-activated, and non-selective cation channel expressed in a variety of cell types including neurons, pancreatic β cells, cardiomyocytes, and immune cells including monocytes/macrophages and neutrophils. The unique characteristic feature of TRPM2 is its temperature sensitivity (Togashi et al., 2006), and the temperature threshold decreases from around 47°C in the absence of oxidative stress to around 35.5°C in the presence of 100 μM H_2_O_2_ (Kashio et al., 2012). Thus, TRPM2 contributes to body temperature regulation (Song et al., 2016; Tan and McNaughton, 2016). TRPM2 is also involved in a variety of physiological functions including immunological cell responses, insulin secretion, and oxytocin release (Kashio and Tominaga, 2017; Szollosi, 2021).

#### TRPM7

TRPM7 is first cloned by three separate groups (Nadler et al., 2001; Runnels et al., 2001; Yamaguchi et al., 2001) and is a constitutively active, homo-tetrameric, non-selective cation channel with protein serine/threonine kinase activity (Nadler et al., 2001; Runnels et al., 2001; Schmitz et al., 2003). The TRPM7 channel is expressed in almost all tissues, including brain, heart, liver, kidney, lung, and spleen (Nadler et al., 2001; Runnels et al., 2001). Accumulating evidence has shown that TRPM7 is essentially involved in a variety of fundamental physiological cell functions, as listed below: cell viability and growth (Nadler et al., 2001; Hanano et al., 2004; Elizondo et al., 2005; Che et al., 2014), cell adhesion (Clark et al., 2006; Su et al., 2006), cytoskeletal regulation (Nadler et al., 2001; Clark et al., 2006), cell migration (Wei et al., 2009; Kuras et al., 2012), cellular and systemic Mg^2+^ homeostasis (Schmitz et al., 2003; He et al., 2005; Ryazanova et al., 2010; Mittermeier et al., 2019), permeation of trace metal ions into cells (Nadler et al., 2001; Monteilh-Zoller et al., 2003; Schmitz et al., 2003), neurotransmitter release (Krapivinsky et al., 2006; Brauchi et al., 2008), axonal growth (Turlova et al., 2016), and the activation and differentiation of immune cells (see Review: Nadolni and Zierler, 2018).

### Biophysical properties of TRPM2 and TRPM7

The monomers of chanzymes TRPM2 and TRPM7 have largely common domain structures, these tetramers both operate as Ca^2+^-permeable cation channels with different biophysical properties, as summarized below.

#### TRPM2

**FIGURE 1:**
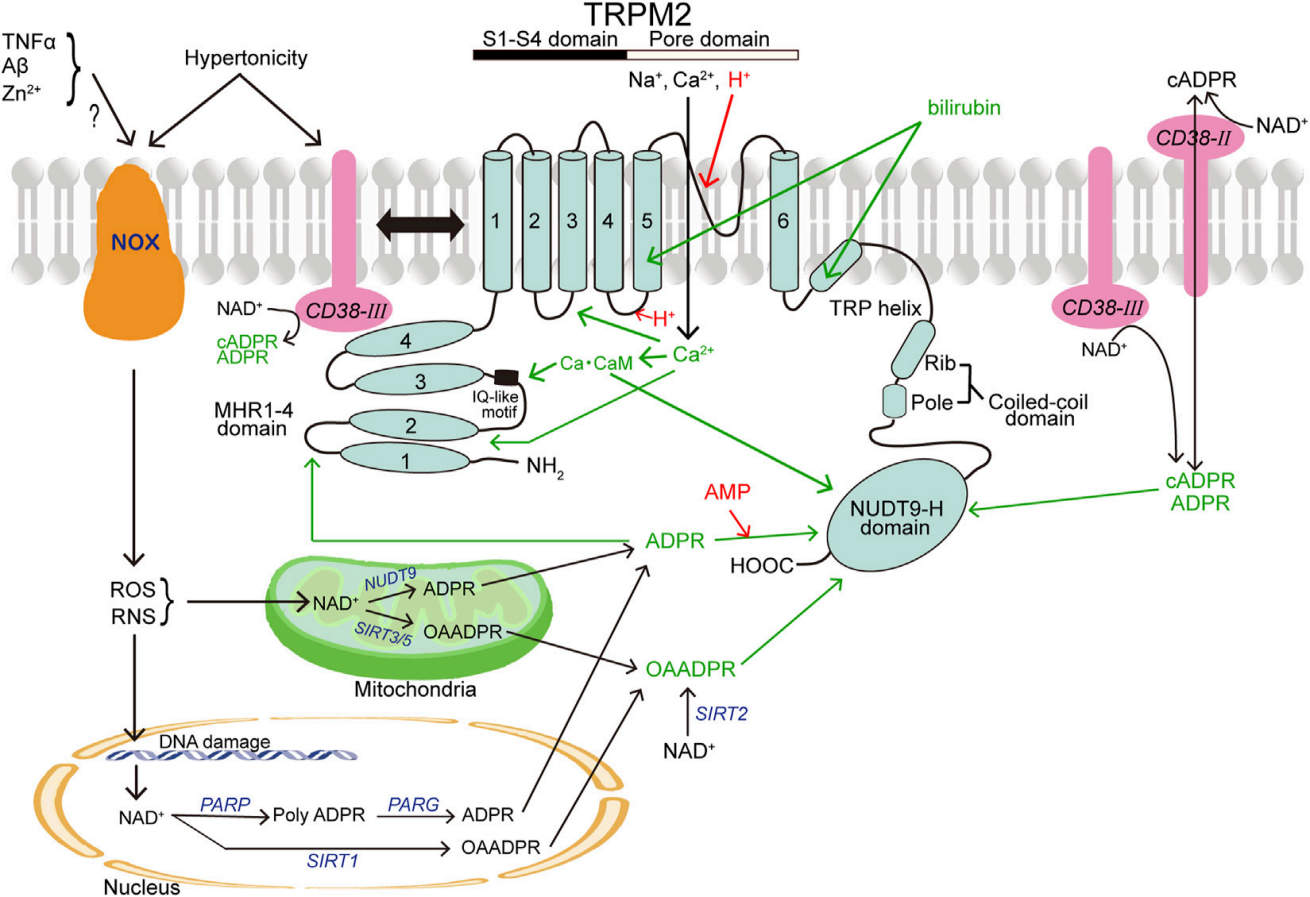
Topology model of a monomeric subunit of TRPM2 channel and its modulators. CB38 (painted in pink) and NOX (in brown) represent two other membrane-spanning proteins that are involved in activation of TRPM2 (in light blue). The names of TRPM2 activators and inhibitors are written in green and red, respectively. In light of the involvement in production of TRPM2 activators, a mitochondrion and a nucleus are also depicted. (See the text for details.)

TRPM2 has MHR1-4 with the IQ-motif at the large *N*-terminus, conserved six transmembrane segment (S1-S6) regions, and a pore-forming loop domain between S5 and S6 as well as the *C*-terminus composed of the TRP helix containing TRP box1 and TRP box2, a coiled-coil domain (CCD), and a unique enzymatic NUDT9 homology (NUDT9-H) domain (Figure 1). The IQ-like motif, located at amino acids (AA) 406–416 of MHR1-4 in human TRPM2 (hTRPM2), is important for Ca-CaM binding (Tong et al., 2006). An additional Ca-CaM-binding motif, W1355-I1368, was found in the NUDT9-H domain of hTRPM2 (Gattkowski et al., 2019). In the pore region, the FGQI motif (AA 979–982) was recently identified as the selectivity filter of hTRPM2 channel (Yu et al., 2021). The CCD is critical for the heteromeric assembly formation of TRPM2 (Mei et al., 2006). In addition, TRPM2 contains a structure of ADP-ribose (ADPR) pyrophosphatase enzyme, although this domain of hTRPM2 is catalytically inactive (Iordanov et al., 2016), and is activated by cytosolic ADPR (cADPR) and reactive oxygen species (ROS) (Hara et al., 2002). Recently, it became clear that ADPR binds not only to the *C*-terminal NUDT9-H domain (AA 1197–1503 for hTRPM2) but also to MHR1-2 located at amino acids 1–422, and it was revealed that the channel activity is greatly affected by the latter binding (Huang et al., 2018b; Huang et al., 2019).

Human TRPM2 exhibits a rapid inactivation time course after activation even in the persistent presence of activators (Csanády and Törocsik, 2009), whereas sea anemone *Nematostella vectensis* TRPM2 (nvTRPM2) does not show such inactivation kinetics (Zhang et al., 2018). The existence of electroneutral residues of G984 and Y985 in the putative selectivity filter of hTRPM2 is judged to be responsible for the inactivation time course, based on the following observations: First, hTRPM2 currents became non-inactivating, when these residues were replaced with acidic residues (G984D and Y985E) (Tóth and Csanády, 2012). Second, the corresponding residues (D1041 and E1042) for an ancient type of nvTRPM2 are acidic or negatively charged (Iordanov et al., 2019).

The TRPM2 single-channel current exhibits a unitary conductance of about 50–80 pS and a linear current-voltage (*I-V*) relationship. The whole-cell current also shows a linear *I-V* relationship. This channel has permeabilities not only to monovalent cations such as Na^+^, K^+^ and Cs^+^ but also to divalent cations such as Ca^2+^ and Mg^2+^ with the permeability ratios to a monovalent cation Na^+^ of 0.67–0.9 and 0.47–0.5, respectively (Kraft et al., 2004; Sano et al., 2001; Xia et al., 2008). Such prominent divalent cation permeabilities are attained by interaction with glutamate, glutamine, and aspartate residues (E960, Q981, D987, and E1022 for hTRPM2) (Belrose and Jackson, 2018; Sumoza-Toledo and Penner, 2011; Turlova et al., 2018; Xia et al., 2008), the residues of which are forming the selectivity filter in the vestibule of the TRPM2 channel pore region (AA 952-1022 for hTRPM2; AA 949-1019 for mouse TRPM2).

#### TRPM7

**FIGURE 2:**
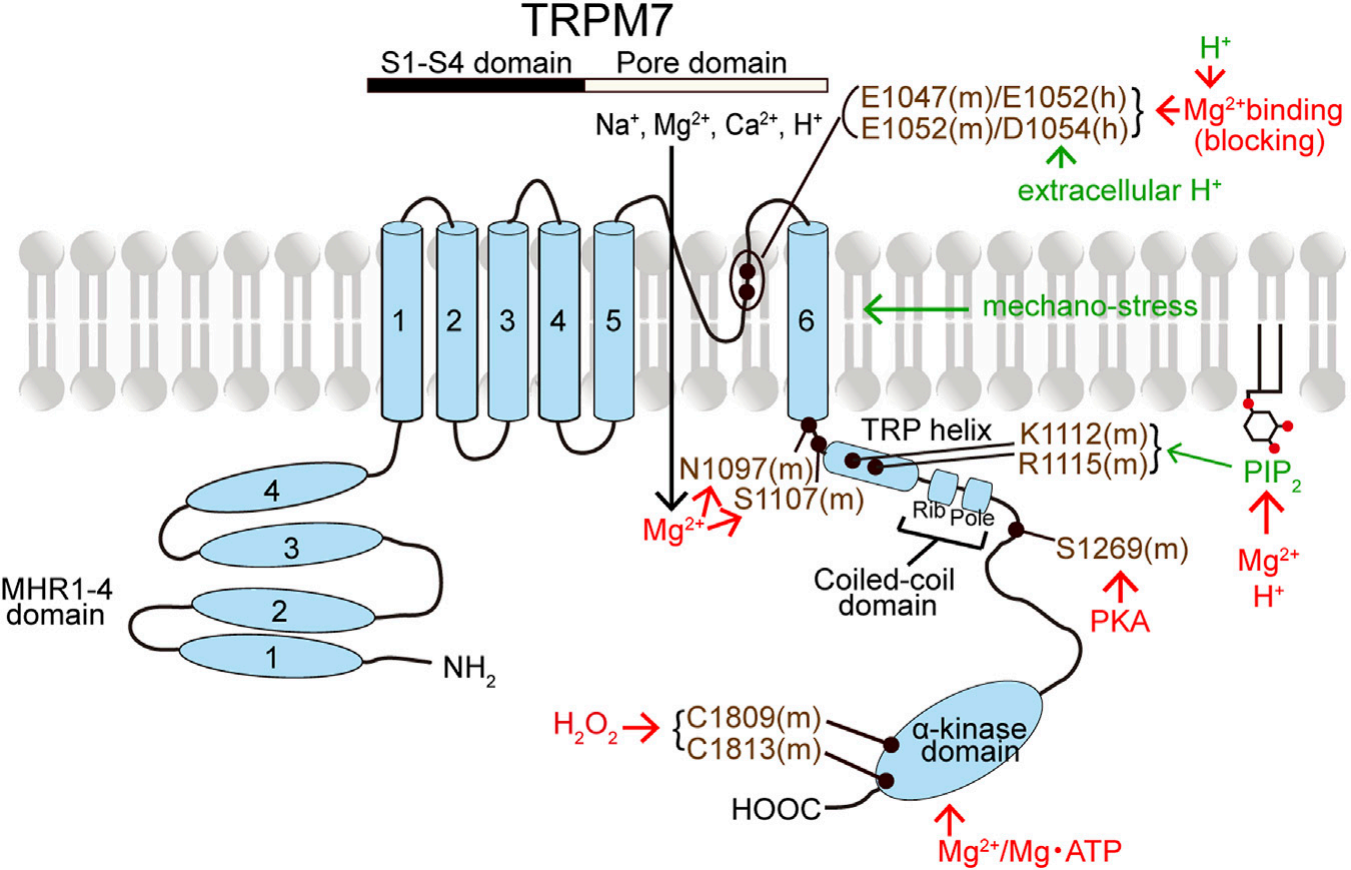
Topology model of a monomeric subunit of TRPM7 channel and its modulators. The names of TRPM7 activators and inhibitors are written in green and red, respectively. TRPM7 residues written in violet represent the putative action sites of TRPM7 activators and inhibitors. (See the text for details.)

TRPM7 has MHR1-4 at the large *N*-terminus followed by the conserved six transmembrane segment (S1-S6) regions containing the pore-forming loop domain between S5 and S6 and the *C*-terminus (Figure 2). The *C*-terminus contains the TRP helix, CCD, and a unique α-kinase domain with an Mg^2+^‧ATP-binding site (Simon et al., 2013; Yee et al., 2014). TRPM7’s characteristic α-kinase activity and its channel function are shown to be independent of each other by site-directed mutagenesis analyses combined with biochemical and electrophysiological studies (Schmitz et al., 2003; Demeuse et al., 2006). On the other hand, the role of α-kinase activity is, in a manner independent of ion channel activity, involved in phosphorylation of downstream signaling molecules such as annexin A1 (Dorovkov and Ryazanov, 2004), myosin II (Clark et al., 2006; Clark et al., 2008a; Clark et al., 2008b), SMAD2 (Romagnani et al., 2017), and PLCγ2 (Deason-Towne et al., 2012) as well as some store-operated Ca^2+^ entry (SOCE) components related to STIM or Orai, thereby regulating SOCE (Faouzi et al., 2017).

The single-channel current of TRPM7 has a unitary conductance of around 40 pS at negative voltages (of around −70 mV) in the absence of extracellular Mg^2+^ (Kozak et al., 2005; Li et al., 2006). The whole-cell currents show a nearly linear *I-V* relationship with weak inward rectification in the absence of extracellular divalent cations, but a non-linear *I-V* relationship with strong outward rectification in the presence of extracellular divalent cations (Nadler et al., 2001; Runnels et al., 2001; Aarts et al., 2003; Monteilh-Zoller et al., 2003; Schmitz et al., 2003) and especially in the presence of extracellular Mg^2+^ due to its open channel blocking action (Kerschbaum et al., 2003). The residues of D1054 and E1052 within the pore-forming region of human TRPM7 provide the binding sites and the selective filter for Mg^2+^ and Ca^2+^ (Numata and Okada, 2008a). On the other hand, in the case of mouse TRPM7, E1047 and E1052 were shown to represent the selectivity filter for Mg^2+^ and Ca^2+^ (Li et al., 2007). Thus, TRPM7 exhibits permeabilities both to monovalent cations such as Na^+^ and Cs^+^ and to divalent metal cations such as Mg^2+^, Ca^2+^, Ba^2+^, Sr^2+^, Cd^2+^, and Zn^2+^. TRPM7 localized in intracellular vesicles mediates Zn^2+^ release from the vesicles upon ROS-induced TRPM7 activation (Abiria et al., 2017). TRPM7 is characteristic in its permeability to protons (Jiang et al., 2005; Numata and Okada, 2008b). The channel permeability to cations including divalent cations is initiated by attraction to the glutamate and aspartate residues, which are negative charges in the pore vestibule of the TRPM7 channel (Li et al., 2007; Numata and Okada, 2008a; Schlingmann et al., 2007; Yee et al., 2014). The TRPM7 channel exhibits inward proton conductance (Jiang et al., 2005) in a manner sensitive to the presence of extracellular Mg^2+^ and Ca^2+^ (Numata and Okada, 2008b). It appears that the proton conductivity of TRPM7 is mediated by the pore *per se*, because the charge-neutralizing mutation of Asp-1054 (D1054A) of human TRPM7 abolished its proton conductance (Numata and Okada, 2008b).

### Activation and inactivation signals for TRPM2 and TRPM7

TRPM2 and TRPM7 channel activities are stimulated or inhibited by numbers of intracellular signaling molecules that are generated or mobilized in response to various environmental stresses, as detailed below.

#### TRPM2

Adenosine diphosphate ribose (ADPR) is a key gating molecule of the TRPM2 channel (Perraud et al., 2001) with an EC_50_ of 10–15 μM (Kolisek et al., 2005; Beck et al., 2006) and exerts the action through the binding not only to the *C*-terminal NUDT9-H domain of TRPM2 (Wehage et al., 2002; Heiner et al., 2003; Perraud et al., 2003; Kraft et al., 2004; Kolisek et al., 2005; Fliegert et al., 2017b) but also to the *N*-terminal MHR1/2 (Huang et al., 2018b; Huang et al., 2019) (Figure 1). A site-directed mutagenesis study showed that hydrogen bonding of R1433 and Y1349 in the *C*-terminus is required for hTRPM2 activation induced by the *C*-terminal binding of ADPR (Fliegert et al., 2017b). For the *N*-terminal ADPR-binding site, M215, Y295, and R302 were identified as the key residues (Huang et al., 2019). In response to stimulation by extracellular signals, especially oxidative stress (reactive oxygen species: ROS) and nitrosative stress (reactive nitrogen species: RNS), ADPR is generated from NAD^+^ in the cytosol via a special type of CD38 (see below), mitochondria via mitochondrial NUDT9 (Perraud et al., 2003; Perraud et al., 2005), and nuclei through the sequential actions of nuclear enzyme poly-ADPR polymerase (PARP) and poly-ADPR glycohydrolase (PARG) in response to DNA damage and PARP stimulation (Caiafa et al., 2009; Esposito and Cuzzocrea, 2009; Fauzee et al., 2010) (Figure 1). TRPM2 is also activated by ADPR upon stimulation with other extracellular stimuli including TNFα in mouse cardiac ventricular myocytes (Roberge et al., 2014), amyloid β-peptide (Aβ) in rat striatal cells (Fonfria et al., 2005), and Zn^2+^ in mouse microglial cells (Mortadza et al., 2017). ADPR-induced TRPM2 activation depends on the presence of phosphatidylinositol 4,5-bisphosphate (PIP_2_) in the inner leaflet of cell membrane (Tóth and Csanády, 2012; Zhang et al., 2018) and cytosolic Ca^2+^ (Du et al., 2009a; McHugh et al., 2003; Starkus et al., 2007; Tong et al., 2006). ADPR cannot activate TRPM2 in the absence of Ca^2+^ (McHugh et al., 2003; Starkus et al., 2007) and of calmodulin (CaM) (Tong et al., 2006). CaM directly interacts with the IQ-like motif (AA406-416) in the *N*-terminus of TRPM2 (Tong et al., 2006) as well as with the Trp-1355∼Ile-1368 motif in the NUDT9-H domain of hTRPM2 with a Kd of 110 nM (Gattkowski et al., 2019) (Figure 1). Intracellular Ca^2+^ alone was also reported to activate TRPM2 in a manner independent of ADPR and the ADPR-binding site of the *C*-terminus of TRPM2 by Du et al. (Du et al., 2009a), although such was not observed by other groups (Csanády and Törocsik, 2009; McHugh et al., 2003; Starkus et al., 2007). In any case, not only ADPR binding but also Ca^2+^ binding are prerequisite for TRPM2 channel gating (Csanády and Törocsik, 2009). By making site-directed mutagenesis combining with patch-clamp functional assay, it was suggested that the D267-D268 motif in the *N*-terminus as a Ca^2+^-binding EF-loop is also critical for hTRPM2 channel activation induced by an unrealistically high concentration (50 mM) of Ca^2+^ (Luo et al., 2018). In addition, cryo-EM study recently revealed that the Ca^2+^-binding site is located at the intracellular border of the channel in between S2, S3, and the TRP helix coordinated by E843 and Q846 of S2, N809 of S3, and E1073 of the TRP helix of hTRPM2 (Wang L. et al., 2018). ROS and RNS were shown to stimulate the TRPM2 channel in intact cells (Hara et al., 2002; Wehage et al., 2002) in an indirect manner through promotion of ADPR generation (Fonfria et al., 2004; Perraud et al., 2005; Buelow et al., 2008; Blenn et al., 2011) (Figure 1). However, it is noteworthy that a splice variant of TRPM2 with deletion of the *C*-terminus can still be activated by H_2_O_2_ (though not by ADPR), suggesting that there exists some ADPR-independent activation mechanism. Of note, H_2_O_2_ reduces temperature thresholds for TRPM2 activation, thereby inducing TRPM2 activation at body temperature (37°C), in a manner independent of ADPR (Kashio et al., 2012). In connection with this, exposure to H_2_O_2_ was shown to induce tyrosine phosphorylation of TRPM2 with activation of TRPM2 channels (Zhang et al., 2007), though the detailed mechanism is not clarified as yet. TRPM2 is also known to be activated by nicotinamide dinucleotide (NAD^+^) (Sano et al., 2001; Hara et al., 2002; Wehage et al., 2002) and by its metabolite, nicotinic acid adenine dinucleotide phosphate (NAADP) (Beck et al., 2006) as well as by cADPR (Fleig and Penner, 2004; Mei et al., 2006; Perraud et al., 2001; Xia et al., 2008). However, NAD^+^-induced TRPM2 activation observed previously (Sano et al., 2001; Hara et al., 2002; Naziroğlu and Lückhoff, 2008) is now considered to be due to contamination with ADPR or metabolism of NAD^+^ (Beck et al., 2006; Grubisha et al., 2006), because after affinity-purification, NAD^+^ and NAADP were found to be incapable of stimulating TRPM2 even at concentrations considerably higher than their cytosolic concentrations (Tóth et al., 2015). In contrast, cADPR can directly gate the TRPM2 channel at high concentrations (Kolisek et al., 2005) with an EC_50_ of 60–120 μM (Beck et al., 2006) and therefore seems to exert as a non-physiological low affinity agonist (Yu et al., 2019). *O*-acetyl-ADP ribose (OAADPR), which is generated in response to ROS/RNS by protein deacetylase sirtuins in the cytosol (via SIRT2), mitochondria (via SIRT1), and nuclei (via SIRT3/5), can also directly activate TRPM2 by binding to the NUDT9-H domain (Grubisha et al., 2006; Tong and Denu, 2010) (Figure 1). TRPM2 activity involves a multifunctional single-pass transmembrane glycoprotein enzyme, CD38, which converts NAD^+^ and NAAD into cADPR and then hydrolyzes cADPR to ADPR (Kim et al., 1993; Takasawa et al., 1993; Zocchi et al., 1993). Also, it is noted that CD38 is not only a generator of cADPR and ADPR but also a transmembrane transporter of cADPR and ADPR (Franco et al., 1998; Guida et al., 2004), thereby mediating the intracellular actions of cADPR and ADPR generated extracellularly (Figure 1). The most widely known type of CD38 (Type II) is ectoenzyme with the catalytic domain facing outside. In recent years, it was shown that there exists another type of CD38 (Type III) with an opposite orientation of which the catalytic domain is facing the cytosol, thereby catalyzing the synthesis of intracellular cADPR (Liu et al., 2017; Zhao et al., 2012). We showed, for the first time, that TRPM2 physically interacts to CD38, and activity of the ∆*C*-variant of TRPM2 (TRPM2-∆C) functioning as a hypertonicity-induced cation channel (HICC) is regulated by this direct interaction between TRPM2 and CD38 (Numata et al., 2012). Indeed, the CD38-TRPM2 interaction was shown to play important roles in oxytocin secretion (Higashida et al., 2018), natural killer cell activity (Rah et al., 2015), and chronic inflammation (García-Rodríguez et al., 2018; Balinas et al., 2019). Also, it is noted that hypertonic stimulation induces ROS production in kidney cells (Ikari et al., 2013; Yang et al., 2005) as well as HeLa cells (Numata et al., 2012) and a marked increase in the intracellular cADPR concentration (Numata et al., 2012). Moreover, both cationic currents activated by hypertonicity and by cADPR were inhibited not only by TRPM2-siRNA but also by CD38-siRNA (Numata et al., 2012). Thus, it is concluded that hypertonic stress induces increases in intracellular cADPR, presumably via activation of CD38, thereby activating the TRPM2 channel as HICC (Figure 1). 2′-Deoxy-ADPR, which is an endogenous nucleotide synthesized from nicotinamide mononucleotide (NMN) and deoxy-ATP by consecutive action of an NMN adenylyl transferase and CD38, was identified as an additional agonist of TRPM2 (Fliegert et al., 2017a), although its binding site is not identified as yet. One type of growth factors, vascular endothelial growth factor (VEGF), was shown to activate TRPM2 thereby inducing Ca^2+^ influx in vascular endothelial cells leading to cadherin phosphorylation (Mittal et al., 2015) and angiogenesis (Negri et al., 2019). Most recently, bilirubin and its derivatives were found to activate TRPM2 channels from the extracellular side by directly interacting with K928 and D1069 existing in the S5 and TRP helix regions of TRPM2, respectively (Figure 1), presumably after getting into an intramembrane deep cavity surrounded by the S3, S5 and TRP helix (Liu et al., 2023).

TRPM2 channel activity is sensitive to extracellular and intracellular acidification. Du et al. (Du et al., 2009b) found that extracellular protons inhibit TRPM2 with an IC_50_ of pH 5.3 by interacting at H958, D964, and E994 existing in the outer vestibule of the TRPM2 pore, whereas Yang et al. (Yang et al., 2010) found that extracellular proton-induced TRPM2 inhibition (with IC_50_ of pH 4.7) is mediated by binding to several residues in the outer vestibule of the pore especially K953 and D1002. In contrast, Starkus et al. (Starkus et al., 2010) reported that extracellular acidification inhibits TRPM2 with an IC_50_ of pH 6.5 after permeating the TRPM2 pore and interacting with an intracellular site. Intracellular protons were observed to completely suppress TRPM2 activity at pH 6 by competitively antagonizing intracellular Ca^2+^ binding by the latter group (Starkus et al., 2010) and with an IC_50_ of pH 6.7 by interacting at D933 in the S4-S5 linker region thereby decreasing sensitivity to intracellular Ca^2+^ and/or intracellular ADPR by the former group (Du et al., 2009b) (Figure 1). AMP, which is a breakdown product of ADPR, is known to specifically antagonize ADPR-induced activation of TRPM2 with an IC_50_ of 10–70 μM (Kolisek et al., 2005; Beck et al., 2006; Lange et al., 2008) through a competition for the Nudix domain (Figure 1). A most abundant trace metal, Zn^2+^, inactivates the channel activity of TRPM2 overexpressed in HEK293 cells, in a manner dependent on membrane potentials, by the extracellular application (Yang et al., 2011). Zn^2+^-induced suppression was found to be full for the inward currents but only partial for the outward currents, suggesting an open-channel blocking action. Positively charged Lys^952^ and negatively-charged Asp^1002^ in the outer pore region may provide the blocking site of Zn^2+^, because charge-neutralizing mutations of these residues (K952A and D1002A) were observed to strongly attenuate the Zn^2+^-induced suppression (Yang et al., 2011). On the other hand, another trace metal, Cu^2+^, inhibited, in a manner independent of voltages, hTRPM2 currents with an IC_50_ of 2.6 μM, when applied extracellularly but not intracellularly (Zeng et al., 2012). However, Cu^2+^ was found to fail to affect the activity of mouse TRPM2 (mTRPM2), in which the residue corresponding to H995 of hTRPM2 is Q992, though the inhibitory effect of Cu^2+^ on hTRPM2 was bolstered (Yu et al., 2014). In fact, H995 was found to be critical for Cu^2+^-induced hTRPM2 inactivation, because charge-neutralizing mutation of His^995^ (H995A or H995Q) in the pore-forming region abolished the Cu^2+^-induced suppression (Yu et al., 2014).

#### TRPM7

The constitutive activity of TRPM7 channel is maintained by PIP_2_ (Runnels et al., 2002; Kozak et al., 2005; Gwanyanya et al., 2006). Thus, TRPM7 currents are inactivated by PIP_2_ depletion caused by PLC-coupled GPCR stimulation (Runnels et al., 2002; Langeslag et al., 2007) and by expression of voltage-sensitive phosphatase (VSP) (Xie et al., 2011). PIP_2_ is known to bind to cationic residues of some TRPs, including TRPV1, TRPM8, and TRPM4 (Rohács et al., 2005; Bousova et al., 2015; Poblete et al., 2015). Similarly, K1112 and R1115 existing in the TRP domain were suggested to be required for PIP_2_ dependence of mouse TRPM7 (Figure 2), because TRPM7 currents were found to be ablated by the K1112Q/R1115Q double mutation (Xie et al., 2011). In contrast to the inhibitory effect of PIP_2_ depletion, transient PIP_2_ hydrolysis was rather found to augment TRPM7 currents (Langeslag et al., 2007).

Intracellular free Mg^2+^ and Mg‧ATP suppress, in a manner independent of voltages, TRPM7 channel activity (Nadler et al., 2001; Runnels et al., 2001; Hermosura et al., 2002; Kozak and Cahalan, 2003; Schmitz et al., 2003). Depletion of intracellular Mg^2+^ or Mg‧ATP augments TRPM7 currents (Nadler et al., 2001; Kozak and Cahalan, 2003; Demeuse et al., 2006; Langeslag et al., 2007). Intracellular free Mg^2+^ completely inhibits TRPM7 currents at millimolar concentrations (Nadler et al., 2001) and suppresses the currents in a dually concentration-dependent manner with two independent sites. Such dual Mg^2+^-induced inhibitory effects were thus described by two IC_50_ values of 10–25 and 90–165 μM in Jurkat T lymphocytes (Chokshi et al., 2012b; Chokshi et al., 2012c) or of 5.6–6.5 and 467–558 μM in TRPM7-overexpressing HEK293 cells (Inoue et al., 2021; Inoue et al., 2014). Extracellular Mg^2+^ also inhibits TRPM7 currents (Nadler et al., 2001) in a manner dependent on voltages with IC_50_ values of 3.2 μM at −40 mV and 0.11 mM at +80 mV (Numata et al., 2007b), indicating voltage-dependent Mg^2+^ block of the TRPM7 channel pore. Intracellular Mg‧ATP inhibits TRPM7 activity with an IC_50_ of 2 mM (Demeuse et al., 2006). Sensitivity to intracellular Mg^2+^ and Mg‧ATP may be mediated by *C*-terminal sites (Figure 2), one within and another outside the kinase domain (Schmitz et al., 2003; Demeuse et al., 2006; Yu H. et al., 2013). The kinase activity is not essential for the TRPM7 channel activity, because mutation of two autophosphorylation sites or of a key catalytic site that abolished kinase activity never affected the channel activity (Matsushita et al., 2005). However, the interaction between the kinase domain and channel domain is involved in the modulation of channel activity by altering the sensitivity to Mg^2+^ and Mg‧ATP (Demeuse et al., 2006; Yu H. et al., 2013). In fact, recently, it was clarified that the channel domain-kinase domain interaction increases TRPM7 currents by decreasing Mg^2+^-induced inhibition (Inoue et al., 2021). In this study, after cleaving the kinase domain, the channel domain (AA 1-1509) *per se* was found to be sensitive to intracellular Mg^2+^ with an IC_50_ of 3.0 μM, and the interaction of the channel domain with the kinase domain was shown to rather decrease intracellular Mg^2+^ sensitivity (Inoue et al., 2021). Based on the truncation studies, the CCD of zebrafish TRPM7 (drTRPM7) was suggested to be involved in the channel’s regulation by Mg^2+^ and Mg‧ATP (Jansen et al., 2016). An involvement of the inter-subunit region between S6 and the TRP domain in the intracellular Mg^2+^ sensitivity of TRPM7 gating was also suggested, because the S1107E mutant of mouse TRPM7 (mTRPM7) exhibited constitutively active channels in a manner insensitive to intracellular Mg^2+^ (Hofmann et al., 2014). Indeed, the Mg^2+^-insensitive S1107E mutant of mTRPM7 was shown to become less sensitive to PIP_2_ depletion (Zhelay et al., 2018). Recently, N1097 of mTRPM7 was also indicated to form the intracellular Mg^2+^ regulatory site, because the N1097Q mutation abrogated the inhibition of TRPM7 channel by physiological intracellular Mg^2+^ concentration (Schmidt et al., 2022). In addition, intracellular Mg^2+^ was reported to inhibit TRPM7 channel activity by screening the negatively charged PIP_2_ (Kozak et al., 2005) and thereby disrupting the PIP_2_-TRPM7 interaction (Zhelay et al., 2018) (Figure 2). Recently, cAMP/PKA was shown to downregulate the TRPM7 activity and expression by phosphorylating TRPM7 at S1269 existing near the CCD region (Tian et al., 2018; Broertjes et al., 2019) (Figure 2). Furthermore, TRPM7 channel activity was demonstrated to be inhibited by ADP-ribosylation factor-like GTPase 15 (ARL15) through forming a macromolecular complex together with TRPM7 and cystathione-β-synthase (CBS)-pair domain divalent metal cation transport mediator (CNNM) (Kollewe et al., 2021; Mahbub et al., 2023).

TRPM7 currents are activated by cytosolic alkalinization and inactivated by intracellular acidification (Kozak et al., 2005). Cytosolic protons inhibit TRPM7 channel with an IC_50_ of pH 6.3 (Chokshi et al., 2012c) by a charge screening of PIP_2_, thereby disrupting the PIP_2_-TRPM7 interaction (Kozak et al., 2005; Zhelay et al., 2018). In contrast, extracellular acidification potentiates TRPM7 presumably by proton-induced unbinding of Ca^2+^ and Mg^2+^, thereby removing the blocking effects of Ca^2+^ and Mg^2+^ (J. Jiang et al., 2005), at the divalent cation binding sites, the negatively charged E1047 and E1052 for mTRPM7 (Li et al., 2007), and E1052 and D1054 for human TRPM7 (hTRPM7) (Numata and Okada, 2008a) in the pore region (Figure 2). In fact, hTRPM7 activation by extracellular protons was abolished by electro-neutralizing D1054 mutation, but not charge-preserving D1054E mutation (Numata et al., 2019).

TRPM7 was shown to be activated by ROS under anoxic conditions (Aarts et al., 2003). Such an enhancing effect of ROS may be caused by increased expression of TRPM7 mRNA and protein in cells exposed to oxidant agents (Wuensch et al., 2010; Nuñez-Villena et al., 2011). In contrast, recently, increased extracellular H_2_O_2_ concentrations were found to inhibit TRPM7 currents in a manner dependent on Mg^2+^ (with an IC_50_ of 16 μM) but not on ATP (Inoue et al., 2014). The Mg^2+^-insensitive S1107E mutant of mTRPM7 is not affected by H_2_O_2_ (Inoue et al., 2021). Therefore, it is likely that the inhibitory effect of H_2_O_2_ is based on the enhancement of intracellular Mg^2+^ sensitivity. Supportively, C1809 and C1813 locating in the kinase domain of mTRPM7, which are essential not only for the Mg^2+^ sensitivity but also for kinase activity (Runnels et al., 2001), were shown to exert as the oxidative stress sensor in the presence of intracellular Mg^2+^ (H. Inoue et al., 2021).

Mechano-stress is an additional activating factor for TRPM7. Under the whole-cell recordings, osmotic cell swelling and shear stress induced by perfusion of bath solution were found to augment Mg^2+^-sensitive cation currents in hTRPM7-transfected, but not mock-transfected, HEK293 cells (Numata et al., 2007a). The shear stress-induced augmentation of whole-cell TRPM7 current was not affected by an exocytosis-blocking reagent brefeldin A under the experimental conditions employed in this study, although laminar flow-induced shear stress was reported to cause exocytotic translocation of TRPM7 to the plasma membrane in some cell types (Oancea et al., 2006). It is noteworthy that even under the cell-free inside-out configuration, membrane stretch was found to directly activate single-channel activity of TRPM7 by increasing the open probability (*P*_*o*_) (Numata et al., 2007a). Moreover, similar Mg^2+^-sensitive single-channel and whole-cell cation currents were also observed upon application of membrane stretch and hypotonic stress, respectively, in human epithelial HeLa cells, in which TRPM7 is endogenously expressed, in a manner sensitive to siRNA-mediated knockdown of TRPM7 (Numata et al., 2007b). In contrast, it was suggested that TRPM7 senses the osmotic gradient rather than membrane stretch in HEK293 cells transfected with hTRPM7, on the basis of observations that whole-cell currents were only slightly increased by cell ballooning induced by intracellular pressure application during observation of TRPM7 activity under the whole-cell configuration (Bessac and Fleig, 2007). In this study, however, there remains a possibility that whole-cell TRPM7 currents were largely pre-activated by cell swelling caused by oncotic pressure due to the cytosolic presence of considerable amount of non-diffusible large organic (colloidal) osmolytes under the experimental conditions where the intracellular pipette solution and the extracellular bath solution had the identical osmolarity. Also, swelling-activated and shrinkage-inhibited Cl^−^ currents might have been, at least in part, contaminated in the whole-cell currents recorded under Cl^−^-rich conditions. The fact that TRPM7 directly senses mechanical stimulation was, in fact, confirmed in human bone marrow mesenchymal stem cells by measuring suction-induced and hydrostatic pressure-induced membrane currents (Xiao et al., 2015) and in HEK293 cells transfected with mTRPM7 by observing pressure-induced cytosolic Ca^2+^ increases (R. Zhao et al., 2019). Mechano-sensitivity of TRPM7 was also shown by Ca^2+^ imaging in mouse mesenchymal stroma cells in response to fluid shear stress (Liu et al., 2015), in rat odontoblasts in response to hypotonic stimulation (Won et al., 2018), and in human MDA-MB-231 adenocarcinoma cells in response to a hydrostatic pressure increase (Zhao et al., 2019). The mechano-sensitivity of TRPM7 might be implicated in hypertension, since molecular TRPM7 expression in vascular smooth muscle cells was found to be decreased in spontaneously hypertensive rats (Touyz et al., 2006).

### Pharmacological properties of TRPM2 and TRPM7

For TRPM2 and TRPM7, a large number of potent antagonists have been identified, but only several agonists were found, as listed below. However, most of them are not so specific to TRPM2 or TRPM7. Most of the binding sites for antagonists and agonists of TRPM2 and TRPM7 await future identification.

#### TRPM2

Since ADPR and cADPR are endogenous activators of TRPM2 channels, it is quite natural that TRPM2 activity is inhibited by ADPR analogs, 8-bromo-ADP-ribose (8-Br-ADPR), at 900 μM (Partida-Sanchez et al., 2007), 8-phenyl-2′-deoxy-ADPR with an IC_50_ of 3 μM (Moreau et al., 2013), and Compound 7i and 8a at 5–6 μM (Luo et al., 2018) as well as by a cADPR analog, 8-bromo-cyclic ADP-ribose (8-Br-cADPR), at ≥100 μM (Kolisek et al., 2005; Beck et al., 2006) by antagonizing the binding of ADPR and cADPR. Recently, 8-Br-cADPR was shown to exert an inhibitory action by binding not to the *C*-terminal binding site, the NUDT9-H domain, but to the *N*-terminal binding site, the U-shaped MHR1/2 domain (Huang et al., 2019). PARP inhibitors, SB750139-13, PJ34, and DPQ, are also effective to suppress TRPM2 activity with IC_50_ values of 25.1 nM, 2.0 μM, and 15.8 μM, respectively (Fonfria et al., 2004). TRPM2 inhibition was observed to be abolished by exposure to a cell-permeable peptide targeting the Nudix motif of TRPM2, tat-M2NX, at 100 μM (Shimizu et al., 2016). Hydroxyl radical scavengers, dimethylthiourea (DMTU) and *N*-2-mercaptopropyonyl glycine (MPG), were shown to inhibit TRPM2 activity (Smith et al., 2003; Ishii et al., 2006). However, it must be noted that these chemicals exert inhibitory actions not directly to TRPM2 but indirectly via scavenging hydroxyl radicals. A Janus kinase 2 (JAK2) inhibitor tyrphostin, AG490, was also found to indirectly antagonize, in a manner independent of JAK2, TRPM2 channel activity by scavenging hydroxyl radicals (Shimizu et al., 2014). AG490-related compounds, AG555 and AG556, blocked H_2_O_2_-induced activation of TRPM2 channels more strongly than AG490 (Toda et al., 2016). A known phospholipase A2 (PLA2) inhibitor, *N*-(*p*-amylcinnamoyl) anthranilic acid (ACA), also blocks TRPM2 currents, in a manner independent of inhibition of PLA2, when applied extracellularly (but not intracellularly), with an IC_50_ of 1.7 μM (Kraft et al., 2006). However, it must be noted that ACA is not specific for TRPM2 but is also known to inhibit activities of TRPM8 and TRPC6 (Kraft et al., 2006). Recently, one of the derivatives of ACA, called compound A23, was found to be a more effective and selective blocker for TRPM2 with an IC_50_ of 788 nM (Zhang et al., 2021).

TRPM2 currents are blocked by an antipyretic acid-derivative nonsteroidal anti-inflammatory drug (NSAID), flufenamic acid (FFA), at 50–1000 μM with an IC_50_ of 70 μM (Hill et al., 2004a). FFA also inhibits HICC/TRPM2-∆C activity (Wehner et al., 2003b) with an IC_50_ of 117 μM (Numata et al., 2007c). However, it must be noted that FFA affects not only TRPM2 but also other ion channels including some chloride, sodium, potassium and calcium channels (Guinamard et al., 2013). FFA analogs, mefenamic acid (MFA) and niflumic acid (NFA), are also effective to inhibit TRPM2 channels with IC_50_ values of 76 and ∼120 μM, respectively (Chen et al., 2012). Another fenamate analog, 2-aminoethoxydiphenyl borate (2-APB), suppresses TRPM2 activity with an IC_50_ of 1.2 μM (Togashi et al., 2008) and HICC activity with an IC_50_ of 525 μM (Numata et al., 2007c). 2-APB is not specific for TRPM2, because it also inhibits other TRPM members (Togashi et al., 2008) including TRPM7 (Li et al., 2006) and multiple TRPC channels (Xu et al., 2005) but activates several TRPV channels (Hu et al., 2004). In addition, a natural plant-derived polyphenol, curcumin, was more recently found to inhibit TRPM2 channels with an IC_50_ of around 50 nM (Kheradpezhouh et al., 2016). However, curcumin has been shown to inhibit a variety of ion channels such as K^+^ channels, Ca^2+^ channels, CFTR, and VSOR/VRAC Cl^−^ channels (Zhang et al., 2014). Furthermore, TRPM2 activity is sensitive to antifungal agents, clotrimazole and econazole, with IC_50_ values of 3–30 μM (Hill et al., 2004b) as well as miconazole with IC_50_ of <3 μM (Togashi et al., 2008). However, these antifungal agents are not specific TRPM2 blockers but are known to block TRPV5 (Nilius et al., 2001), Ca^2+^-activated IK channels (Jensen et al., 1998), ATP-sensitive K^+^ channel (Jäger et al., 2004), and L-type Ca^2+^ channels (Thomas et al., 1999).

In contrast to these TRPM2 inhibitors, an analgesic and antipyretic drug, acetaminophen, was found to activate TRPM2 in rat hepatocytes at high concentrations (10–15 μM) (Kheradpezhouh et al., 2014).

#### TRPM7

TRPM7 inhibitors are divided into five categories: 1. *In vivo* metabolites such as sphingosine and spermine. 2. Natural products such as waixenicin A, carvacrol, and ginsenoside-Rd (GS-Rd) as well as quinine. 3. Non-specific channel blockers including NS8593, SKF-9635, and 2-APB. 4. Enzyme antagonists such as nafamostat, CCT128930, 5-ligoxygenase (5-LOX) inhibitors, and TG100-115. 5. Anesthesia-related drugs including lidocaine and midazolam.

Sphingosine is an amino alcohol forming a cell membrane phospholipid and potently inhibits TRPM7 currents with an IC_50_ of 590 nM (Qin et al., 2013). Sphingosine also blocks TRPM6 (IC_50_ of 460 nM) but neither TRPM2 nor TRPM4. Its structural analog fingolimod FTY720, which is an immunosuppressant and the first oral drug for treatment of multiple sclerosis, blocks TRPM7 with an IC_50_ of 720 nM (Qin et al., 2013). These facts may suggest their inhibiting actions are mediated by interacting with membrane phospholipids. Spermine is a tetravalent cationic polyamine and can block, in a voltage-dependent manner, TRPM7 channel activity with an IC_50_ of 2.3 μM from the extracellular side (Kozak et al., 2002) but not by the intracellular application (Kerschbaum et al., 2003). Single substitution of Ser-1107, which is known to be the site for sensitivity to intracellular Mg^2+^ (Hofmann et al., 2014), of TRPM7 by Glu (S1107E) was found to reduce the sensitivity not only to Mg^2+^ but also to spermine (Zhelay et al., 2018). Thus, it is conceivable that spermine-induced inhibition of TRPM7 activity is, in a manner similar to intracellular Mg^2+^, mediated by electrostatic screening and resultant disruption of interaction between PIP_2_ and TRPM7 (Zhelay et al., 2018).

Several natural products have been shown to block TRPM7 channels. Among them, the most potent and specific blocker is waixenicin A which is a xenicane diterpenoide derived from marine soft coral (Zierler et al., 2011). Waixenicin A inhibits, in a manner dependent on the intracellular Mg^2+^ concentration ([Mg^2+^]_i_), TRPM7 with IC_50_ values of 16 nM and 7 μM in the presence and absence of 0.7 mM [Mg^2+^]_i_, respectively. Since K1648R mutation of Mg^2+^-binding site on the kinase domain increased the IC_50_ value to 2.5 μM in the absence of [Mg^2+^]_i_, it is suggested that intracellular Mg^2+^ facilitates the binding of waixenicin A to TRPM7 (Zierler et al., 2011). Waixenicin A is exceptionally selective to TRPM7 against other TRP channels including TRPM6 (Zierler et al., 2011; Beesetty et al., 2018) and even against zebrafish TRPM7 (Jansen et al., 2016). TRPM7 is inhibited by carvacrol, which is a monoterpene phenolic compound derived from plant volatile oils, with an IC_50_ of 306 μM (Parnas et al., 2009). GS-Rd isolated from kampo herbal medicine ginseng was shown to inhibit TRPM7 with an IC_50_ of 170–178 μM (Kim, 2013). A plant alkaloid quinine, which is isolated from the bark of a cinchona, is used to treat malaria and a known blocker for Ca^2+^-activated K^+^ channels, can effectively inhibit TRPM7 channels at 30 μM (Chubanov et al., 2012).

A non-selective cation channel blocker NS8593, an aminobenzimidazole derivative, inhibits TRPM7 currents, in a manner sensitive to intracellular Mg^2+^, with IC_50_ values of 1.6 and 5.9 μM in the absence and presence of 0.3 mM [Mg^2+^]_i_, respectively (Chubanov et al., 2012). Since the mutation of Tyr-1049 on the pore-forming loop (Y1049P) resulted in reduction of IC_50_ values to 0.47 and 1.9 μM in the absence and presence of 0.3 mM [Mg^2+^]_i_, respectively, the TRPM7 pore loop is likely to be involved in the interaction between NS8593 and TRPM7 (Chubanov et al., 2012). NS8593 was shown to be selective at 10 μM to TRPM7 against other TRPs including TRPM2, TRPM3, TRPM5, TRPM8, TRPC6, TRPV1, and TRPA1 (Chubanov et al., 2012). Another non-selective cation channel blocker, 2-APB, inhibits TRPM7 channels with an IC_50_ of 178 μM but enhances TRPM6 channels with an EC_50_ of 205 μM (Li et al., 2006). Its inhibiting action is not direct to TRPM7 but indirect through an intracellular acidification (Chokshi et al., 2012a). 2-APB also inhibits the other multiple TRP members especially TRPM2, as mentioned in the above section. A classical non-selective cation channel blocker SKF-96365 exhibits irreversible full inhibition of TRPM7 at 20 μM (Kozak et al., 2002). However, it is noted that SKF-96365 can block voltage-dependent T-type Ca^2+^ channels as well (Singh et al., 2010).

Some blockers for a variety of enzymes have been shown to block TRPM7. A synthetic broad-spectrum serine protease inhibitor, nafamostat, inhibits TRPM7 currents in a manner dependent on voltages and on extracellular divalent cations (Chen X. et al., 2010). In this study, the IC_50_ values were found to be 15 μM at −100 mV and 121 μM at +100 mV in Ca^2+^- and Mg^2+^-free bathing solution, whereas those values increased to 514 μM at −100 mV and 617 μM at +100 mV in the presence of extracellular 1 mM Ca^2+^ and Mg^2+^ in TRPM7-transfected HEK293 cells. Nafamostat-induced inhibition was largely depressed by the charge-neutralizing mutation of Glu-1052 (E1052A) (Chen X. et al., 2010). Thus, it is concluded that Glu-1052 is one of negatively charged residues important for inhibition of TRPM7 channels not only by divalent cations (Numata and Okada, 2008a) but also by nafamostat. Endogenous TRPM7 expressed in mouse hippocampal neurons was inhibited by nafamostat with an IC_50_ of 27 μM in the presence of a low concentration (0.1 mM) of Ca^2+^ and Mg^2+^ in a bath solution, whereas the channels were unexpectedly found to be augmented by pre-application of nafamostat (Chen X. et al., 2010). A potent AKT inhibitor CCT128930 preferentially blocks, in an Mg^2+^-independent manner, TRPM7 with an IC_50_ of 0.63–0.86 μM, compared to TRPM6 and TRPM8, presumably by interacting with multiple residues in the selectivity filter (Guan et al., 2021). 5-LOX inhibitors such as NDGA, AA861, and MK886, were also found to inhibit TRPM7 channel activity, in a manner independent of the effects on 5-LOX, at 10–20 μM (Chen H. C. et al., 2010). In addition, TG100-115, which is a PI3Kγ/δ inhibitor, was found to inhibit not only the TRPM7 kinase activity with an IC_50_ of 1.07 μM but also the TRPM7 channel activity (Song et al., 2017).

Several anesthetic drugs were identified to be effective inhibitors of TRPM7 channels. Local anesthetic lidocaine inhibits TRPM7 currents with an IC_50_ of 11.1–11.6 mM in a voltage-independent and frequency-dependent manner (Leng et al., 2015). A widely used clinical anesthetic benzodiazepine, midazolam, can suppress TRPM7 currents by treatment for seconds and also inhibit TRPM7 expression by treatment for 48 h (Chen J. et al., 2016).

In addition to these TRPM7 inhibitors belonging to five categories, VER155008, which is an adenosine-derivative inhibitor of heat shock protein 70 (Hsp70), was recently shown to potently suppress TRPM7 channel activity in a manner independent of the kinase activity without affecting TRPM2, TRPM3, TRPM6, and TRPM8 channels (Rössig et al., 2022).

A δ-opioid receptor antagonist, naltriben, was found to be an effective activator of TRPM7 (Hofmann et al., 2014). Naltriben voltage-independently activated TRPM7 channels with an EC_50_ of 20.7 μM in a manner independent of intracellular Mg^2+^ and competitive with NS8593, but had no effect on TRPM2, TRPM8, and TRPV1 channels. The site of naltriben action is most likely localized in or near the TRP domain, because the S1107E mutant, which is a constitutively active channel insensitive to intracellular Mg^2+^, became insensitive to naltriben. A benzimidazole compound, milbefradil, which displays structural homology to benzimidazole NS8593, activated TRPM7-mediated Ca^2+^ entry with an EC_50_ of 53 μM and activated TRPM7 currents at 100 μM with a physiological intracellular Mg^2+^ concentration (0.9 mM) but failed to activate with a higher [Mg^2+^]_i_ (1.8 mM), whereas milbefradil failed to activate TRPM3, TRPA1, and TRPV1 channels (Schäfer et al., 2016). This study also showed that the S1104E mutation of TRPM7 became insensitive to melbefradil. However, it must be pointed out that milbefradil inhibits voltage-dependent T-type Ca^2+^ channels (Bezprozvanny and Tsien, 1995; Viana et al., 1997) and volume-sensitive VSOR/VRAC Cl^−^ channels (Nilius et al., 1997b) as well.

### Three-dimensional structures of TRPM2 and TRPM7

In the last several years, the three-dimensional structures of TRP channels have become well elucidated owing to the studies with applying cryo-electron microscopy (cryo-EM), as recently reviewed (Cao, 2020; Huffer et al., 2020; Zubcevic, 2020). Here, such cryo-EM structures of TRPM2 and TRPM7 are shortly summarized below.

#### TRPM2

**FIGURE 3:**
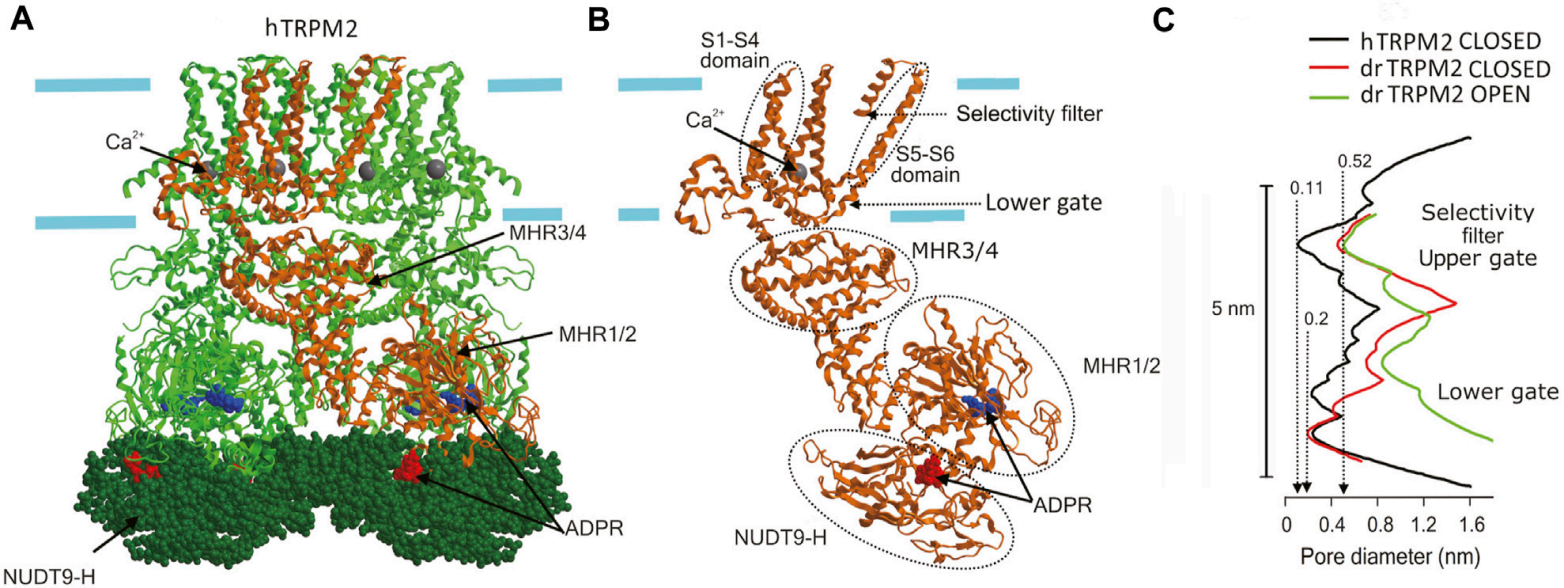
Cryo-EM structure of TRPM2. **(A)** The structure of hTRPM2 viewed parallel to the membrane plane. The drawing is based on the 6PUS.pdb file downloaded from https://www.rcsb.org/structure/6PUS. **(B)** Single subunit [depicted in **(A)** in brown] showing the structural elements mentioned in the text. **(C)** The pore radius along the central axis for drTRPM2 in the closed (apo, red line) and open (ADPR/Ca^2+^-bound, green line) states (Huang et al., 2018b) and for hTRPM2 reconstituted into the lipid nanodiscs in closed (black line) state (Yu et al., 2021). Approximate positions of the selectivity filter (upper gate) and the lower gate [also shown in **(B)**] are indicated.

The three-dimensional structure of TRPM2 channel has been established by cryo-EM (Huang et al., 2020; Szollosi, 2021) using orthologous recombinant proteins from starlet sea anemone *N. vectensis* (nvTRPM2) (Zhang et al., 2018), zebrafish *Danio rerio* (drTRPM2) (Huang et al., 2018b; Yin et al., 2019), and human *Homo sapience* (hTRPM2 or hsTRPM2) (Huang et al., 2019; Wang L. et al., 2018; Yu et al., 2021). Like other six-transmembrane domain cation channels, TRPM2 proteins formed homotetramers with an overall shape reminiscent of a square prism or a bell with a height of up to 16 nm and a large cytosolic part (∼80%) (Figure 3A). The structure of the voltage sensor-like domain (formed by S1-S4) and of the pore domain (formed by S5-S6) resembles that of other voltage-gated cation channels (Figure 3B). However, unlike voltage-gated K^+^ channels, in TRPM2 (like in other TRPs), the voltage sensor-like domain lacks the conserved array of positively charged arginine and lysine and interacts with the S5-S6 of the adjacent subunit in a domain-swapping manner (Cao, 2020; Xia et al., 2019). Thus, TRPM2 is largely insensitive to voltage. The transmembrane portion of the pore is ∼5 nm long. It begins with an external vestibule with a diameter of ∼1 nm (for nvTRPM2) lined by a double ring of negative charges. The vestibule is followed by a short (∼1 nm) selectivity filter with a constriction of ∼0.52 nm in diameter, which was mostly invariant for all orthologues viewed in detergent micelles (Figure 3C). However, the ligand-free hTRPM2 reconstituted into the lipid nanodiscs had much narrower and ion-impermeable selectivity filter of ∼0.11 nm (Figure 3C), suggesting that it may serve as an upper gate which allowed passage of cysteine-modifying Ag^+^ only in the open but not in the closed state (Yu et al., 2021). The open TRPM2 pore is large enough to pass hydrated Na^+^ ions and tetramethylammonium but not *N*-methyl-*D*-glucosamine (NMDG). At around 3.5 nm from the entrance is located the gate which was interpreted as a lower gate by Yu et al. (Yu et al., 2021) formed by the S6 helices. Here, the pore is too tight (diameter is less than 0.2 nm for all orthologues in the closed state) to pass even a single water molecule. In the open state of the drTRPM2 bound to Ca^2+^ and ADP-ribose (ADPR), the lower gate widens up to a diameter of ∼0.9 nm allowing passage of hydrated Na^+^ and Ca^2+^ ions. This gate is followed by an internal vestibule, which is also negatively charged but narrower (diameter of 0.47 nm for nvTRPM2) compared to the external entrance. After passing the inner vestibule, ions come to the cytoplasmic cavity that has upper and lower chambers. This part is best described for the nvTRPM2 (Zhang et al., 2018). A bent tunnel with a diameter of over 0.4 nm connects the upper chamber of the cytoplasmic cavity with the Ca^2+^-binding site. This site is located near the membrane-cytosol interface but outside the central pore axis (Figure 3A). It is formed mostly by the ends of S2 helices and is accessible not only from the cytoplasmic cavity but also from the outside the protein via peripheral tunnels. Thus, the cytosolic parts of the channel form a porous structure with a complex system of cavities. The cytosolic Ca^2+^ ions first reach the Ca^2+^-binding site via peripheral tunnels moving nearly parallel to the membrane surface, then come to the upper cytoplasmic cavity, and only then may access the selectivity filter when the gate is open. Although a calmodulin-mediated mechanism was suggested for TRPM2 activation by cytosolic Ca^2+^ (Du et al., 2009a), mutations of the negatively charged amino acids at the Ca^2+^-binding site (E893A in nvTRPM2) of the channel protein itself greatly reduced or even abolished Ca^2+^-induced activation (Zhang et al., 2018), suggesting a key role of Ca^2+^ coordination within the Ca^2+^-binding pocket in the channel activation mechanism. ADPR, an obligate co-activator, binds to the U-shaped MHR1/2 and to the *C*-terminal NUDT9-H domain, which is unique for TRPM2 and is homologous to the mitochondrial ADPR pyrophosphatase NUDT9 (Huang et al., 2019; Huang et al., 2020; Szollosi, 2021) (Figures 3A, B). Based on the previous finding that nvTRPM2 but not hTRPM2 is activated by ADPR even after deletion of the NUDT9-H domain (Kühn et al., 2016) and on the comparison between the ADPR-binding sites of sea anemone, zebrafish, and human orthologues, it is speculated that the ADPR-binding site could be evolutionarily shifted from the MHR1/2 to the NUDT9-H domain of TRPM2 protein (Fliniaux et al., 2018) and that MHR1/2 could bind cADPR, which synergistically enhances the effect of ADPR (Fliegert et al., 2020). The ROS-dependent increase in ADPR production may link TRPM2-mediated Ca^2+^ influx to the inflammasome activation (Wang et al., 2020). MHR3/4 domains link the agonist-sensing regions (NUDT9-H and MHR1/2) with the pore and thus transduces chemical activation signals to the channel opening (Huang et al., 2018b; Huang et al., 2020). PIP_2_ binding is also necessary for TRPM2 channel activity and is thought to occur near the Ca^2+^-binding site (Huang et al., 2020; Szollosi, 2021; Zhang et al., 2018), although its exact site is undetermined. Molecular and structural nature of the temperature sensing by TRPM2 awaits future elucidation, although a role of a dynamic protein-membrane lipid relationship was suggested as the general concept (Zubcevic, 2020).

#### TRPM7

**FIGURE 4:**
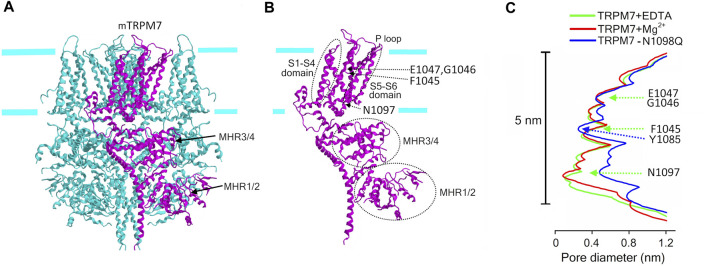
Cryo-EM structure of TRPM7. **(A)** The structure of mTRPM7 viewed parallel to the membrane plane. The *C*-terminal protein kinase domain is truncated in this structure. The drawing is based on the 5ZX5. pdb file downloaded from https://www.rcsb.org/structure/5ZX5. **(B)** Single subunit (depicted in (A) in magenta) showing the structural elements mentioned in the text. **(C)** The pore radius along the central axis for mTRPM7 in the presence of EDTA (green line) and high Mg^2+^ (red line) and for the constitutively active N1098Q mutant (blue line) (Duan et al., 2018; Nadezhdin et al., 2023). Indicated amino acids mark the selectivity filter (F1045, G1046, E1047) with the Mg^2+^-binding site formed by G1046 and E1047, and the lower gate (N1097), and the narrowest point of the pore of open-state channel (Y1085).

Unlike TRPM2, TRPM7 is an Mg^2+^-permeable channel, which, in addition, possesses the protein kinase domain which is enzymatically active. The cryo-EM structure of the slightly truncated mTRPM7 protein fused to the maltose binding protein revealed an overall similarity to other TRP channels (Figure 4A) in terms of tetrameric assembly and orientation of helices (Duan et al., 2018; Huang et al., 2020). The tight lower gate at the cytoplasmic end of S6 (N1097) is similar to that of the TRPM2. The conduction pathway did not change upon removal of Mg^2+^ and other divalent cations (Figure 4C). The Mg^2+^-binding site is formed by the negative charge of Glu (E1047 for mTRPM7) and backbone carbonyl of Gly (G1046 for mTRPM7), which along with F1045 form the selectivity filter within the pore region (P-loop) of S5-S6 (Figure 2; Figure 4B). This region also contains a disulfide bond, which is stabilized by Mg^2+^ and important for regulation by glutathione. The structure of the mouse TRPM7 resolved by Duan et al. (Duan et al., 2018) did not contain the *C*-terminal kinase domain which possesses cysteine residues (C1809 and C1813 for mTRPM7) important for the second low-affinity Mg^2+^-binding site and for TRPM7 channel inhibition by oxidative stress (Inoue et al., 2021; Inoue et al., 2014) (Figure 2). Very recently, Nadezhdin et al. (Nadezhdin et al., 2023) analyzed cryo-EM structures of constitutively activated mTRPM7 channels with using the gain-of-function N1098Q mutant (Schmidt et al., 2022) and wild type mTRPM7 stimulated with a potent TRPM7 activator, naltriben. This study showed that the open-state of TRPM7 channel is associated with an increase in the pore size at the lower gate (near N1097) to ∼0.23 nm and turning of the tyrosine (Y1085) hydroxyls towards the ion conducting pathway thereby forming the narrowest point of the pore (Figure 4C). This study also identified a binding site for highly potent inhibitors, VER155008 and NS8593, which stabilize the closed conformation of TRPM7 channel. Although the cryo-EM structure of the kinase domain of mouse TRPM7 is still missing, this structure was previously revealed by X-ray crystallography and found to be similar to classical protein kinases such as PKA (Yamaguchi et al., 2001).

## Roles of TRPM2 and TRPM7 in cell volume regulation and cell death induction/protection

### Roles of TRPM2 and TRPM7 in cell volume regulation

Animal cells must cope with fluctuations of osmotic gradient across the cell membrane by two types of cell volume regulation mechanisms: cell volume recovery after osmotic shrinkage called RVI and that after osmotic swelling called RVD (see Reviews: Hoffmann et al., 2009; Okada, 2004; Wehner et al., 2003a). After hypertonic and hypotonic challenges, animal cells attain RVI and RVD mainly by net gain of most abundant extracellular small osmolytes Na^+^ and Cl^−^ and by net loss of most abundant intracellular small osmolytes K^+^ and Cl^−^, respectively, with accompanying fluxes of osmotically obligated water, thereby readjusting the intracellular osmolarity to the extracellular osmolarity. In these volume regulation processes, TRPM2 and TRPM7 play important roles in animal cells.

#### RVI and TRPM2

RVI is known to be accomplished by Na^+^-conductive hypertonicity-induced cation channels (HICCs) and/or Na^+^-permeable electroneutral transporters such as Na^+^/H^+^ antiporter (NHE), Na^+^-K^+^-Cl^−^ symporter (NKCC) and Na^+^-Cl^−^ symporter (NCC) (see Reviews: Hoffmann et al., 2009; Okada, 2004). Among them, HICC was shown to be the most effective mechanism of RVI (Wehner et al., 2006; Plettenberg et al., 2008). To attain volume-regulatory NaCl influx, parallel activation of some unidentified type of anion channel, tentatively labelled the hypertonicity-induced anion channel (HIAC) (Okada, 2016), is to be required. Such HIAC-like currents were notably observed in human hepatoma HepG2 cells (Bondarava et al., 2009).

**FIGURE 5:**
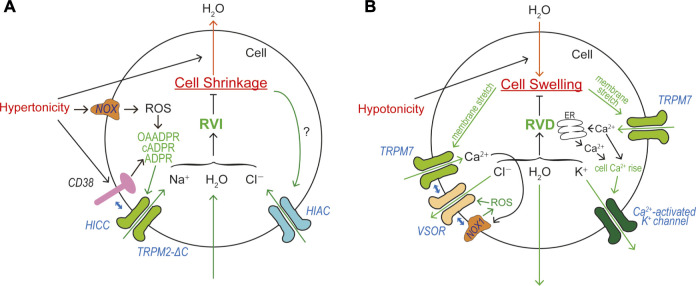
Roles of TRPM2 and TRPM7 in cell volume regulation (CVR). **(A)** Roles of HICC (TRPM2-∆C *plus* CD38) and HIAC in CVR attained after cell shrinkage, RVI. Depolarization caused by HICC activation drives Cl^−^ influx via the HIAC pore. Resultant NaCl influx drives water influx leading to RVI. **(B)** Roles of TRPM7, VSOR, and Ca^2+^-activated K^+^ channel in CVR attained after cell swelling, RVD. Hyperpolarization predominantly caused by Ca^2+^-activated K^+^ channels drives Cl^−^ efflux via the VSOR channel pore. Resultant KCl efflux drives water efflux leading to RVD.

HICC currents were, for the first time, recorded in response to an isotonic challenge in Intestine 407 cells equilibrated under hypotonic conditions, and were suggested to be involved in the post-RVD RVI process (Okada and Hazama, 1989). So far, two groups of HICCs have been reported: the one is amiloride-sensitive and Gd^3+^-insensitive, and another is amiloride-insensitive, Gd^3+^- and/or FFA-sensitive (Wehner et al., 2003a; Wehner et al., 2006). Amiloride-sensitive HICC currents were originally observed in rat hepatocytes and shown to be involved in RVI (Wehner et al., 1995). Molecular correlates for this type of HICC in hepatocytes and HepG2 cells have been suggested to be several members of ENaC (Böhmer and Wehner, 2001; Plettenberg et al., 2008; Bondarava et al., 2009). In addition to δENaC, recently TRPM2 and TRPM5 were also suggested to be implicated in the molecular architecture of HICC in HepG2 (Koos et al., 2018). Gd^3+^-sensitive amiloride-insensitive HICC currents involved in RVI were first found in airway epithelial cells (Chan and Nelson, 1992), and were shown to be also sensitive to FFA in mouse cortical collecting duct M-1 cells (Volk et al., 1995). The molecular entity of this type of HICC was identified as TRPM2-∆C *plus* CD38 in HeLa cells, and the RVI event was shown to be inhibited by knockdown of TRPM2 or CD38 (Numata et al., 2012). The molecular mechanism of TRPM2-mediated RVI is schematically depicted in Figure 5A.

#### RVD and TRPM7

RVD is a prerequisite function for animal cells devoid of the cell wall which is covering plant cells. RVD is attained by net loss of mainly KCl (and partly intracellular small organic osmolytes). Now, activation of separate conductive pathways for K^+^ and Cl^−^ (and some negatively-charged amino acids) is known to be the predominant mechanism in most mammalian cells, although electroneutral KCl transport pathways such as K^+^-Cl^−^ symporter (KCC) are involved in RVD of erythrocytes (Hoffmann et al., 2009; Okada, 2004). First evidence for the fact that volume-regulatory KCl efflux is accomplished by parallel activation of K^+^ and Cl^−^ channels was provided by electrophysiological approaches in epithelial Intestine 407 cells (Hazama and Okada, 1988) and T lymphocytes (Cahalan and Lewis, 1988). Since a large variety of K^+^ channels are installed in the plasma membrane of animal cells, swollen cells exploit some of them as volume-regulatory K^+^ channels depending on cell types (Wilson and Mongin, 2018). In human Intestine 407 cells, a Ca^2+^-activated K^+^ channel was shown to serve as the volume-regulatory K^+^ channel (Hazama and Okada, 1988) and later molecularly identified as IK1 (Wang et al., 2003). For the volume-regulatory conductive Cl^−^ pathway, a new type of Cl^−^ channel was discovered to be activated by cell swelling in Intestine 407 cells (Hazama and Okada, 1988; Kubo and Okada, 1992) and T lymphocytes (Cahalan and Lewis, 1988). In contrast to involvements of various volume-regulatory K^+^ channels, only this type of Cl^−^ channel was shown to predominantly serve as the volume-regulatory Cl^−^ channel in most animal cells. This ubiquitous Cl^−^ channel has been well characterized and called the volume-sensitive outwardly rectifying anion channel: VSOR (Okada, 1997), the volume-regulated Cl^−^ channel: VRAC (Nilius et al., 1997a), or the volume-sensitive organic osmolyte/anion channel: VSOAC (Strange and Jackson, 1995). In 2014, the core molecule of VSOR was identified as LRRC8A (Qiu et al., 2014; Voss et al., 2014), and VSOR activity was shown to additionally require at least one of its paralogs (LRRC8C, LRRC8D, and/or LRRC8E) as the subcomponent of VSOR (Voss et al., 2014).

Essential roles of intracellular Ca^2+^ rise in RVD were shown in many cell types (Cala et al., 1986; Grinstein et al., 1982; Hazama and Okada, 1988; Hazama and Okada, 1990a; Rothstein and Mack, 1990; Wong and Chase, 1986). In the human epithelial cells, swelling was found to induce activation of Ca^2+^-permeable cation channels (Y. Okada et al., 1990) and then triggers Ca^2+^ release from the intracellular Ca^2+^ store and sizable cytosolic Ca^2+^ rise (Hazama and Okada, 1990b), thereby stimulating volume-regulatory Ca^2+^-activated K^+^ channels. Later, the molecular identity of swelling-activated Ca^2+^-permeable cation channel was identified as TRPM7 (Numata et al., 2007b) which exhibits direct mechano-stress sensitivity (Numata et al., 2007a). TRPM7-mediated Ca^2+^ influx is thus involved in RVD (Numata et al., 2007b) by triggering activation of volume-regulatory Ca^2+^-activated K^+^ channel. This Ca^2+^ inflow may also be involved in VSOR activation, because VSOR was shown to be activated by ROS (Browe and Baumgarten, 2004; Shimizu et al., 2004; Varela et al., 2004) through the NOX activation regulated by a local Ca^2+^ rise in the immediate vicinity of open Ca^2+^-permeable cation channels called Ca^2+^ nanodomain (Akita and Okada, 2014). It is noteworthy that NOX1 does physically interact not only with LRRC8A (Choi et al., 2016) but also with LRRC8C and LRRC8D (Choi et al., 2021). Recently, TRPM7 was also demonstrated to physically interact with LRRC8A thereby playing a role as an essential regulator for VSOR expression (Numata et al., 2021). Steady-state Ca^2+^ influx through TRPM7 enhances molecular expression of LRRC8A mRNA. In addition, the plasmalemmal presence of TRPM7 stabilizes the plasma membrane expression of LRRC8A protein through the protein-protein interaction between LRRC8A and the *C*-terminal α-kinase domain of TRPM7. Collectively, TRPM7 is involved in RVD in a multiple fashion: first by mediating swelling-induced Ca^2+^ influx leading to cytosolic Ca^2+^ rise, thereby activating volume-regulatory Ca^2+^-activated K^+^ channels and inducing hyperpolarization driving Cl^−^ efflux through any available Cl^−^ channels; second by mediating formation of the Ca^2+^ nanodomain, thereby activating volume-regulatory VSOR Cl^−^ channels; third by stimulating molecular expression of LRRC8A mRNA in swollen cells; and fourth by stabilizing plasmalemmal expression of LRRC8A through the physical interaction with LRRC8A protein. The molecular mechanism of TRPM7-mediated RVD is schematically illustrated in Figure 5B.

### Roles of TRPM2 and TRPM7 in cell death

In a huge number of publications, both TRPM2 and TRPM7 have been reported to have pathophysiological relevance to cell death and tissue injury. Cell death-inducing machineries utilize not only VSOR/VRAC and ASOR/PAC anion channel activities (see Reviews: Okada et al., 2021a; Okada et al., 2021b) but also the activities of TRPM2 and TRPM7 that are sensor cation channels constitutively expressed. Cell death is classified into apoptosis and necrosis. Necrotic cell death can be distinguished to accidental and programmed necrosis, and the latter one is further sorted into pyroptosis, necroptosis, and ferroptosis. Here, we summarize how TRPM2 and TRPM7 are implicated in these cell death modes. It is noted that both types of TRPM members often exert as double-edged swords in cell death induction.

#### Inductive/protective roles of TRPM2 in cell death and tissue injury

Reactive oxygen species (ROS) are generated in many of pathological conditions such as ischemia/reperfusion (I/R) injury (Granger and Kvietys, 2015) and exposure to pathogenic factors leading to neurodegenerative disorders. These pathogenic factors include amyloid β (Aβ) peptide developing Parkinson’s disease (Hensley et al., 1994), methyl-4-phenyl-1,2,3,6-tetrahydropyridine (MPTP) which is used to induce Alzheimer’s disease model (Wu et al., 2003), inflammatory cytokines, and others. ROS are well known to cause cell death and dysfunction. Pathological roles of ROS-activated TRPM2 channel in cell death have been extensively studied in various tissues and cell types including neuronal cells (see Review: Malko and Jiang, 2020). Hydrogen peroxide, a kind of ROS, has been used to mimic oxidative stress experimental models. The earliest study showed that TRPM2 (the former name, LTRPC2) activation in TRPM2-expressing HEK293T cells by H_2_O_2_ and TNFα leads to the cell death which is dependent on Ca^2+^ influx and is suppressed by a Ca^2+^ chelator and antisense oligonucleotides against TRPM2 (Hara et al., 2002). H_2_O_2_-induced cell death was also reproduced in primary neurons and was found to be attenuated by extracellular Ca^2+^ removal with abolishing TRPM2 activation. The effects of PARP inhibitors and downregulation of TRPM2 by siRNA confirmed an involvement of TRPM2 activation downstream of PARP activity in H_2_O_2_-induced neural death (Fonfria et al., 2005; Kaneko et al., 2006). An involvement of TRPM2 activation in H_2_O_2_-induced cell death was also evidenced by siRNA-mediated knockdown of *Trpm2* in non-neuronal cells, such as immortalized mouse embryonic fibroblasts (Blenn et al., 2011) and mouse RAW264.7 macrophages (Zou et al., 2013). A dominant negative variant of TRPM2 (TRPM2-S) showed an inhibitory effect on cell death mediated by TRPM2 activation (Zhang et al., 2003), and PKC-mediated phosphorylation of TRPM2-S exhibited an inhibitory effect on TRPM2 activation and cell death (Hecquet et al., 2014). TRPM2 is reportedly involved in homeostasis of intracellular Zn^2+^ regulating cell death. H_2_O_2_-treatment of hippocampal neurons caused an intracellular Zn^2+^ increase by lysosomal dysfunction, Zn^2+^ release from lysosome, mitochondrial Zn^2+^ accumulation, mitochondrial fission, and cell death (Li et al., 2017). These H_2_O_2_-induced lysosomal/mitochondrial toxicities in neurons were attenuated by TRPM2 gene knockout and by a Zn^2+^-specific chelator, confirming contribution of TRPM2 and dysregulation of intracellular Zn^2+^ in neural cell death.

Involvements of TRPM2 in cell death induced by I/R or oxygen-glucose deprivation/reoxygenation (OGD/R) were demonstrated by TRPM2 gene knockdown or knockout in brain neurons (Jia et al., 2011; Shimizu et al., 2013; Verma et al., 2012; Ye et al., 2014) and myocardial cells (Hiroi et al., 2013). Bilirubin, which exerts as an direct activator for TRPM2, was most recently found to be released in the brain subjected to oxygen-glucose deprivation (OGD), and to aggravate brain damage in the stroke in a manner strongly sensitive to molecular perturbation of the bilirubin-binding site on TRPM2 (Liu et al., 2023).

Although TRPM2 plays inductive roles in cell death under a large variety of conditions, as summarized above, TRPM2 channel activity has also been reported to participate in protection from cell death under several specified conditions. TRPM2 was observed to protect neuroblastoma SH-SY5Y cells from H_2_O_2_-induced cell death (S. J. Chen et al., 2013) as well as against lung injury induced by lipopolysaccharide (LPS) (Di et al., 2011), I/R-induced adult heart injury (Miller et al., 2013; Miller et al., 2014; Hoffman et al., 2015), doxorubicin-induced death in breast cancer cells (Koh et al., 2015) and in SH-SY5Y cells (Hirschler-Laszkiewicz et al., 2022), and H_2_O_2_- or hyperthermia-induced tissue damage in the sea anemone (Ehrlich et al., 2022). Thus, activation of TRPM2 channels plays not only detrimental or death-inducing but also beneficial or protective roles depending on the cellular conditions.

#### Inductive roles of TRPM2 in apoptotic cell death

The most cases of H_2_O_2_-induced cell death in which TRPM2 is implicated are classified as apoptosis in a variety of cell types, including human monocytic U937 cells (W. Zhang et al., 2006), rat ventricular myocytes (Jiang et al., 2006), and mouse endothelial cells (Hecquet et al., 2014; Sun et al., 2012). TRPM2 was also found to play essential roles in apoptosis induction caused by stimulation with a variety of exogenous pathogens. These pathogens include TNFα in U937 cells (Zhang et al., 2006) and mouse ventricular myocytes (Roberge et al., 2014), interferon-γ (IFNγ) in mouse microglia (Akyuva et al., 2021) and human neuroblastoma SH-SY5Y cells (Güzel et al., 2021), morphine in mouse hippocampal neurons (Osmanlıoğlu et al., 2020), glyceryltrinitrate (GTN) in mouse trigeminal ganglion neurons (Yazğan and Nazıroğlu, 2021), zinc oxide nanoparticle in human brain vascular pericytes (Jiang et al., 2017), anti-malarian drug, hydroxychloroquine, in human retinal pigment epithelial cells (Ertuğrul et al., 2023), and uric acid in human AC16 cardiomyocytes (Wu et al., 2023). Essential roles of TRPM2 channel were also recently shown in apoptotic cell death induced by an anti-cancer drug, doxorubicin (DOX), in rat cardiac cells (Akyuva and Nazıroğlu, 2023; Yıldızhan et al., 2023) as well.

In the brain stroke, TRPM2 was shown to partake in I/R-induced neuronal apoptosis using TRPM2 knockout mice (Alim et al., 2013; Gelderblom et al., 2014). TRPM2 deficiency was found to be protective against such a brain injury by decreasing the ratio of synaptic NMDAR subunit (GluN2A) to extra-synaptic NMDAR subunit (GluN2B) in the hippocampus (Alim et al., 2013) and attenuating immune cell filtration into the ischemic hemisphere (Gelderblom et al., 2014). I/R- or OGD/R-induced apoptosis was also shown to involve TRPM2 activity in the mouse kidney (Gao et al., 2014) and rat pheochromocytoma PC12 cells (Pan et al., 2020). Traumatic brain injury (TBI) was found to upregulate TRPM2 expression in the rat cerebral cortex and hippocampus (Cook et al., 2010) and to cause apoptotic death in rat hippocampal neurons in a manner dependent on ROS production and sensitive to 2-APB (Yürüker et al., 2015).

TRPM2 channels have been demonstrated to play an important role in induction of apoptotic cell death associated with etiology of several major diseases, such as Alzheimer’s disease, diabetes mellitus, and Parkinson’s diseases. Accumulation of amyloid β (Aβ) is causative of Alzheimer’s disease. ROS-induced neuronal cell death following exposure to Aβ was attenuated by a PARP inhibitor and PARP siRNA (Fonfria et al., 2005). TRPM2 also takes part in Aβ-induced pathological conditions including ER-stress, synaptic loss, microglial activation, and age-related memory deficits (Ostapchenko et al., 2015). A critical role of TRPM2 activity was shown in Aβ-induced neuronal apoptosis (Çınar and Nazıroğlu, 2023; Li and Jiang, 2018). In pancreatic islet cells or β cells, activation of TRPM2 channels was found to be involved in apoptosis induced by H_2_O_2_ (Bari et al., 2009; Lange et al., 2009; Manna et al., 2015) and by diabetic stimuli, such as streptozocin (STZ) (Manna et al., 2015) and palmitate (Li et al., 2017). MPTP and its active metabolite, 1-methyl-4-phenylpyridinium ions (MPP^+^), are neurotoxins for dopaminergic neurons and thereby causing Parkinson’s disease (Burns et al., 1983). MPP^+^ was demonstrated to increase TRPM2 expression in the mouse substantia nigra (Sun et al., 2018) and induce ROS elevation and apoptosis in neuronal SH-SY5Y cells differentiated by the addition of retinoic acid for 6 days (Sun et al., 2018). Since increased TRPM2 immunoreactivity and apoptotic cell death in the rat ovary were found to be coupled to ovarian hyperstimulation syndrome (OHSS) (Şanlı et al., 2021), it is possible that TRPM2 is causatively related to apoptosis induction in this disease.

#### Inductive roles of TRPM2 in non-apoptotic cell death

Participation of TRPM2 channels has been reported also in non-apoptotic cell death, including aponecrosis, necrosis, and pyroptosis under certain conditions. Acetaminophen overdose was found to cause activation of TRPM2 currents and induction of necrotic cell death which exhibited some apoptotic features such as DNA fragmentation and membrane blebbing in a manner sensitive to TRPM2 knockdown and knockout (Kheradpezhouh et al., 2014). This cell death mode might be classified into aponecrosis or secondary necrosis (see Step-6 in Figure 3 depicted in Okada et al., 2019). In addition, TRPM2 was reported to be involved in bile acid-induced cell injury in pancreatic acinar cells that simultaneously exhibited both necrotic and apoptotic cell death (Fanczal et al., 2020). In mouse microglial cells, H_2_O_2_ or Zn^2+^, one of damage-associated molecular pattern molecules (DAMPs), was observed to elicit cytosolic Ca^2+^ rise and necrotic cell death, both of which were abolished by TRPM2 knockdown (Mortadza et al., 2017). In mouse mastocytoma P815 cells, farnesyl pyrophosphate (FPP), a mevalonate pathway intermediate, caused necrotic cell death with exhibiting cell swelling and membrane rupture in a manner partially sensitive to TRPM2 knockout (Chen et al., 2021). Physical and functional interactions between TRPM2 and extra-synaptic NMDA receptors were recently found to exacerbate excitotoxic (necrotic) cell death in mouse cortical neurons under ischemic brain injury (Zong et al., 2022a). Pyroptosis is a caspase-1/3-dependent programmed necrosis that exhibits NLRP3 inflammasome activation in association with inflammation (see Reviews: Green, 2019; Jiang et al., 2020; Kolbrink et al., 2020). ROS-induced TRPM2 activation was shown to be involved in NLRP3 inflammasome activation in bone marrow-derived macrophages stimulated by charged liposomes (Zhong et al., 2013), in human leukemia U937 cells exposed to high glucose (Tseng et al., 2016), in microglia stimulated with Aβ (Alawieyah Syed Mortadza et al., 2018; Aminzadeh et al., 2018), and in OGD/R-challenged PC12 cells (Pan et al., 2020). However, no study has addressed the pyroptotic cell death (due to membrane rupture) eventually induced by the *N*-terminus of gasdermin D/E in relation to TRPM2.

#### Inductive roles of TRPM7 in cell death

Since overexpression of TRPM7 in HEK293 cells was found to cause cell death (Nadler et al., 2001; Monteilh-Zoller et al., 2003; Schmitz et al., 2003), it was assumed that TRPM7 channels are somehow involved in cell death induction under pathophysiological conditions. In fact, Aarts et al. (Aarts et al., 2003), for the first time, found that TRPM7 plays an essential role in neuronal cell death caused by OGD. Prolonged OGD increased non-selective cation conductance (I_OGD_) in cortical neurons independently of the activation of glutamate receptor. I_OGD_ was inhibited by a vitamin E derivative, an O_2_^−^ scavenger and a NOS inhibitor suggesting OGD-induced current activation by reactive oxygen/nitrogen species (ROS/RNS). I_OGD_ led to Ca^2+^-overload followed by a further increase of ROS/RNS production and cell death which are inhibited by a TRPM7 blocker, Gd^3+^, and by TRPM7 siRNA. Subsequently, Sun et al. (Sun et al., 2009) demonstrated that suppression of TRPM7 expression by shRNA inhibits delayed neuronal cell death induced by transient global cerebral ischemia in rat hippocampal CA1 neurons, along with protective effects on ischemia-induced neuronal dysfunction such as defect of long-term potentiation and fear/spatial memories. In addition, molecular expression of TRPM7 was found to be upregulated in the hippocampus of rats subjected to I/R and in cultured rat hippocampal neurons during reoxygenation after transient OGD (Jiang et al., 2008). Similar ischemia-induced upregulation of TRPM7 was subsequently observed in rat brain tissues (Zhan et al., 2014; Zhang et al., 2012) and in the mouse brain (Chen et al., 2015). Downregulation of TRPM7 expression was found to be coupled to acquirement of resistance to an anticancer drug, DOX, in colon cancer LoVo cells (Castiglioni et al., 2015). Moreover, TRPM7 activated by Ca^2+^/Mg^2+^ removal was found to induce Zn^2+^ influx, thereby resulting in Zn^2+^ toxicity in mouse cortical neurons (Inoue et al., 2010). In the presence of extracellular Zn^2+^ ions, OGD was found to lead to cell toxicity in cortical neurons in a manner sensitive to Gd^3+^, a TRPM7 blocker, and TRPM7 shRNA.

#### Inductive/protective roles of TRPM7 in apoptotic cell death

Chen X et al. (2010) found that TRPM7 downregulation suppresses cell death induced by staurosporine (STS) and DOX, which are well-known apoptosis inducers, in HEK293 cells. Consecutively, Coombes et al. (Coombes et al., 2011) reported that H_2_O_2_ induces not only PI-positive (necrotic) but also TUNEL-positive (apoptotic) cell death in primary mouse cortical neurons in a manner sensitive to treatment with EGTA or 2-APB. Later, pharmacological studies also suggested that TRPM7 mediates apoptotic neuronal death caused by brain ischemia through modulation of CaMKII, calmodulin, and calcineurin (Turlova et al., 2021). These results strongly suggest that TRPM7 is involved in apoptosis induction under specified conditions. Indeed, Desai et al. (Desai et al., 2012) concluded that TRPM7 plays an inductive role in Fas-mediated apoptosis based on the following observations. TRPM7 KO attenuated Fas-mediated cell death in primary mouse T-cell. TRPM7 gene silencing by shRNA in Jurkat T-cell suppressed TRPM7 currents and PARP cleavage induced by Fas ligand (FasL). The cell death induction by FasL was suggested to be mediated by the channel function of TRPM7 but not the kinase activity of TRPM7 because the kinase-dead TRPM7 mutant (K1646A) exhibited no effect on FasL-induced caspase-3 activation and PARP cleavage, although the kinase domain is cleaved by caspase-8/3 during Fas stimulation. However, the kinase domain of TRPM7 was suggested to facilitate OGD/R-induced apoptosis in primary mouse cortical neurons through interacting with annexin A1 (Zhao et al., 2015). Subsequently, acetaminophen-induced apoptosis in human hepatoma HEPG2 cells was found to be partially suppressed by siRNA of TRPM7, though the inhibitory effect was less prominent than siRNAs for TRPC1, TRPV1, and TRPM2 (Badr et al., 2016). Also, intra-cortical injection of shRNA for TRPM7 was shown to suppress apoptosis in the cerebral cortex of rats subjected to TBI (Xu et al., 2018). In addition, high glucose-induced neuronal apoptosis was shown to be accompanied by molecular and functional upregulation of TRPM7 and suppressed by TRPM7 siRNA (Huang et al., 2018a). Furthermore, gene silencing of *Trpm7* was demonstrated to inhibit apoptosis induced by an NO donor, sodium nitroprusside (SNP), in rat chondrocytes (Ma et al., 2021).

In contrast to the inductive role of TRPM7 in apoptotic processes, several pieces of molecular evidence for its protective role against apoptotic cell death have also been reported. TRPM7 knockdown was found to increase the rate of apoptotic cell death spontaneously observed during culturing the rat basophilic leukemic mast (RBL-2H3) cells (Ng et al., 2012). In human urinary bladder cancer (BCa) cells, apoptotic cell death was observed, *in vitro*, to be facilitated by TRPM7 knockdown (Cao et al., 2016). In this study, injection of antitumor carvacrol was found to reduce TRPM7 activity, thereby suppressing tumor growth in the mouse bladder cancer *in vivo*. Sun and his collaborators recently reported that apoptotic neuronal cell death induced by a Parkinson’s disease-related neurotoxin, MPP^+^, is protected by TRPM7 activation induced by isoproterenol (Sun et al., 2020a) and by TRPM7 overexpression (Sun et al., 2020b) in dopaminergic differentiated neuroblastoma SH-SY5Y cells. Moreover, in their *in vivo* studies, treatment with the neurotoxin, MPTP, was found to cause apoptotic tissue damage together with reduction of the TRPM7 level in mouse substantia nigra pars compacta (SNpc) region (Sun et al., 2020b). Consistently, they also observed increased expression of apoptotic proteins and decreased TRPM7 levels in the samples obtained from the SNpc region of human Parkinson’s disease patients (Sun et al., 2020b). It is warranted to clarify how TRPM7 plays opposite roles in apoptotic processes depending on experimental conditions.

#### Inductive roles of TRPM7 in non-apoptotic cell death

TRPM7 was shown to be activated by extracellular acidification (Jiang et al., 2005; Numata and Okada, 2008a) and involved in acid-induced necrotic cell death (Numata et al., 2019). Extracellular acidification (< pH 6.0) causes a persistent cell volume increase and cell death which were inhibited by TRPM7 downregulation using siRNA in HeLa cells (Numata et al., 2019). TRPM7 D1054A, an acid-activation-deficient mutant, abolished acidification-evoked cell volume increase and cell death, suggesting acid-evoked activation of TRPM7 leads to a necrotic volume increase (NVI) followed by necrotic cell death. TRPM7 is also likely to be implicated in two types of inflammatory programmed necrosis: caspase-1/3-dependent one called pyroptosis and RIPK-dependent one called necroptosis. The involvement of TRPM7 in pyroptosis was suggested by the pharmacological observations, as follows. First, cell swelling-induced release of an inflammatory cytokine, IL-1β, from human macrophages was inhibited by TRPM7 blockers, 2-APB and SKF-96365 (Compan et al., 2012). Second, LPS-induced release of IL-1β, IL-6, and TNFα from mouse small intestinal IEC-6 cells was inhibited by another TRPM7 blocker, NS8593 (Li et al., 2022). However, it must be pointed out that these recent studies examined the roles of TRPM7 in inflammatory responses but not those in eventual necroptotic cell death as yet. On the other hand, TRPM7 was shown to be involved in necroptotic cell death induced by the treatment with a combination of TNFα, Smac mimetic, and z-VAD-FMK (TSZ) in human colonic epithelial HT29 cells (Cai et al., 2014). The shRNA-mediated knockdown protected the cells from TSZ-induced necroptotic membrane rupture and to inhibit TSZ-induced TRPM7 current activation and Ca^2+^ influx.

### Ionic mechanisms of cell death induction/protection involving TRPM2 and TRPM7

Since cell death induction is tightly coupled to dysfunction of cell volume regulation, in which TRPM2 and TRPM7, as already described, play important roles, the ionic mechanisms of cell death induction may, at least in part, involve TRPM2 and TRPM7 channel activities in relation to altered mechanisms of the cell volume regulation.

#### Roles of TRPM2 and TRPM7 in ionic mechanisms leading to apoptotic cell death

**FIGURE 6:**
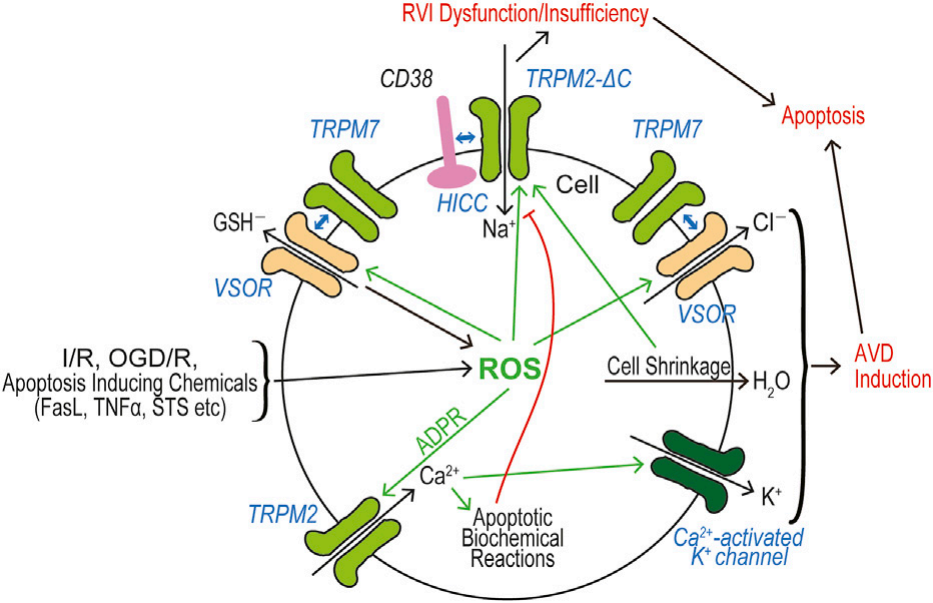
Roles of TRPM2 and TRPM7 in the ionic mechanisms leading to apoptosis induction that requisitely involves AVD induction followed by RVI dysfunction or insufficiency. Hyperpolarization predominantly caused by Ca^2+^-activated K^+^ channels drives effluxes of Cl^−^ and GSH^−^ via the VSOR channel pore. Resultant KCl efflux drives water efflux leading to cell shrinkage. (See the text for details.).

Normotonic cell shrinkage, termed AVD (Maeno et al., 2000), is an earliest and prerequisite event of apoptotic cell death processes (Hasegawa et al., 2012; Maeno et al., 2000; Maeno et al., 2006a; Nukui et al., 2006; Shimizu et al., 2007). The AVD induction is attained by KCl efflux that is accomplished by activation of K^+^ and Cl^−^ channels and drives water efflux (Okada et al., 2001). ROS are produced in response to I/R, OGD/R, and numbers of apoptosis-inducing chemicals and exert as the central mediators linking between apoptotic stimulants and intracellular apoptotic reactions. ROS can activate TRPM2 cation channels (Hara et al., 2002) through ADPR production (Figure 1) and VSOR/VRAC anion channels (Browe and Baumgarten, 2004; Shimizu et al., 2004; Varela et al., 2004) (Figure 6). TRPM2-mediated Ca^2+^ entry not only triggers apoptotic intracellular biochemical reactions (Nicotera and Orrenius, 1998; Orrenius et al., 2003; Rizzuto et al., 2003) but also stimulates Ca^2+^-activated K^+^ channels which are known to be involved in apoptosis induction (see Review: Burg et al., 2006). VSOR plays dual roles in apoptosis induction first by mediating AVD-inducing Cl^−^ efflux and second by mediating antioxidant GSH^−^ efflux driven by membrane hyperpolarization caused by K^+^ channel activation (Figure 6). TRPM7 was recently demonstrated to function as an essential regulator of VSOR by enhancing expression of LRRC8A mRNA via the mediation of Ca^2+^ influx and stabilizing the plasmalemmal expression of LRRC8A through the molecular interaction between LRRC8A and the *C*-terminal domain of TRPM7 (Numata et al., 2021). Thus, TRPM7 may play a facilitating role in apoptosis induction secondary by upregulating VSOR channel activity inducing AVD and by mediating GSH release (Figure 6).

To eventually induce apoptotic cell death, persistent cell shrinkage is to be complemented by RVI dysfunction or insufficiency. In fact, the RVI event was shown to be inhibited during apoptotic processes (Maeno et al., 2006b), through the Akt1 inhibition caused by ROS-induced ASK1 phosphorylation (Subramanyam et al., 2010). In many cell types, RVI is known to be produced by shrinkage-induced NaCl uptake through activation of NHE, NKCC, and/or HICC (Okada et al., 2019). In HeLa cells, the main mechanism of RVI was shown to be activation of HICC (Wehner et al., 2003b), the molecular correlate of which is TRPM2-∆C *plus* CD38 (Numata et al., 2012). Thus, it is possible that HICC activity is downregulated by some intracellular signals produced by apoptotic biochemical reactions, such as phosphorylated ASK1 (pASK1) (Figure 6).

#### Roles of TRPM2 and TRPM7 in ionic mechanisms leading to necrotic cell death

Necrosis is caused by plasma membrane rupture eventually resulted from persistent cell swelling induced by a variety of insults. Necrotic cell death starts with normotonic cell swelling, called NVI (Barros et al., 2001; Okada et al., 2001), which is caused by water uptake driven by influx of osmolytes, chiefly NaCl. Persistence of necrotic cell swelling is accomplished by impairment of RVD (Okada et al., 2004).

**FIGURE 7:**
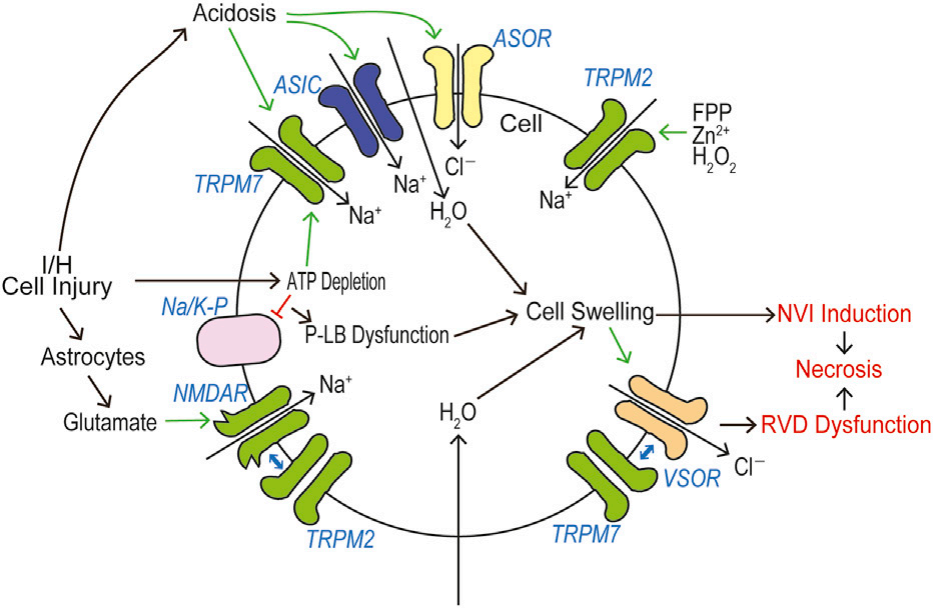
Roles of TRPM2 and TRPM7 in the ionic mechanisms leading to necrosis induction that requisitely involves NVI induction followed by RVD dysfunction. Depolarization caused by activation of TRPM7, ASIC, NMDAR, and TRPM2 drives Cl^−^ influx via the VSOR channel pore. Resultant NaCl influx drives water influx leading to cell swelling. (See the text for details.)

Accidental necrosis is induced by ischemia or hypoxia (I/H) and accidental or traumatic cell injury, because these insults result in ATP depletion within the cells and the resultant extracellular acidosis as well as accumulation of glutamate released from astrocytes in the brain (Okada et al., 2019), as schematically depicted in Figure 7. ATP depletion causes suppression of Na^+^-K^+^ pump (Na/K-P) activity, thereby resulting in oncotic cell swelling due to an impairment of the pump-leak balance (P-LB) mechanism (Okada et al., 2021a; Tosteson and Hoffman, 1960). In addition, ATP depletion augments TRPM7 channel activity (Nadler et al., 2001), thereby causing the influx of cations (mainly Na^+^) and membrane depolarization. Acidosis also stimulates ubiquitous TRPM7 cation channels (Jiang et al., 2005; Numata and Okada, 2008b) and the neuronal amiloride-sensitive acid-sensing ion channel (ASIC) (Waldmann et al., 1997; Waldmann et al., 1999), both of which also bring about Na^+^ influx and membrane depolarization. In addition, acidosis activates ASOR/PAC, which is a ubiquitously expressed anion channel (Okada et al., 2021b). Membrane depolarization elicited by activation of TRPM7 and ASIC cation channels drives Cl^−^ influx via activated ASOR anion channels. Furthermore, in brain neurons, exposure to excessive glutamate stimulates ionotropic glutamate receptors, NMDARs, which operate as non-selective cation channels and produces Na^+^ influx and membrane depolarization (Tymianski, 2011). TRPM2 physically interacts with and functionally augments extra-synaptic NMDARs (Zong et al., 2022a). TRPM2 is also known to be activated by a danger signal FPP (Chen et al., 2021) and H_2_O_2_ or Zn^2+^ (Mortadza et al., 2017). The excessive NaCl entry thus produced by these cation and anion channels drives water influx, causing cell swelling (Figure 7). Such accidental cell swelling involving TRPM2 and TRPM7 may cause necrosis and thus represent NVI induction. In accord with this view, TRPM2 knockout was shown to inhibit necrotic cell death induced by H_2_O_2_ or Zn^2+^ in mouse macrophages (Mortadza et al., 2017), by FPP in mouse mastrocytoma cells (Chen et al., 2021), and by ischemia in mouse neurons (Zong et al., 2022a). Also, acidotoxic necrosis was shown to be abolished by TRPM7 knockdown in HeLa cells (Numata et al., 2019). Cell swelling compelled under such ischemic, acidotoxic, and excitotoxic conditions may activate VSOR anion channels. However, operation of VSOR, the activity of which is much supported by the interaction with TRPM7, cannot serve as a pathway for volume-regulatory Cl^−^ efflux but rather exerts as a pathway for swelling-exaggerating Cl^−^ influx, because depolarization caused by activation of NMDAR, TRPM7, and/or TRPM2 should drive Cl^−^ flux to the inward (but not outward) direction, thereby bringing about the RVD dysfunction (Figure 7). Thus, both TRPM2 and TRPM7 are involved in accidental necrosis by participating in the NVI induction and in the following RVD dysfunction, thus attaining persistent cell swelling due to the continuous inflows of Na^+^, Cl^−^, and osmotically obliged water (Figure 7).

As described in the preceding sections, TRPM2 and TRPM7 were molecularly proved to be involved in pyroptosis and necroptosis, respectively. Swelling- and ROS-activated VSOR/VRAC anion channels were also implicated in pyroptosis (Daniels et al., 2016; Green et al., 2018; Yang et al., 2023; Ye et al., 2021). Depletion or release ATP was observed to be coupled to necroptosis (Leist et al., 1997; Jouan-Lanhouet et al., 2012) and pyroptosis (Yang et al., 2015). Persistent, sizable cell swelling was found to be associated with necroptosis (Chen X. et al., 2016) and pyroptosis (Chen X. et al., 2016; Compan et al., 2012; Fink and Cookson, 2006; Schorn et al., 2011; Yang et al., 2023). Together, it can be assumed that the ionic mechanisms of pyroptosis and necroptotic cells death processes are, at least in part, similar to those of accidental necrosis illustrated in Figure 7. However, no study has as yet been reported as to whether TRPM2 and TRPM7 are involved in another type of programmed necrosis called ferroptosis which is caspase-independent and Fe^2+^-dependent.

## Conclusion and perspective

TRPM2 and TRPM7 are Ca^2+^-permeable non-selective cation channels playing sensor roles for chemical, thermal, and mechanical stimuli. They have unique biophysical, physiological, pharmacological, and structural properties. Both activities are essentially involved as central components of the processes in cell volume regulation/dysregulation and cell death induction/protection not only by conducting cations, mainly Na^+^ and Ca^2+^, but also by interacting with other membrane-spanning proteins including LRRC8A, a core component of VSOR anion channel, NMDAR cation channels, and a cyclic ADP ribose hydrolase, CD38. Taken together, in the last section, it was sought to delve into the ionic mechanisms of cell death induction by focusing altered activities of TRPM2/TRPM7.

TRPM2 and TRPM7 have been shown to be involved in cell death associated with a large variety of diseases such as stroke, Alzheimer’s disease, Parkinson’s disease, and diabetes mellitus, as noted in the previous sections. Besides these, TRPM2 and TRPM7 were reported to be somehow related to many other diseases, although it is not clear whether these cation channels are involved in cell death processes coupled to those diseases. TRPM2 was shown to be implicated in the pathogenesis of epilepsy (Zheng et al., 2020) and atherosclerosis (Zong et al., 2022b). Also, TRPM2 was suggested to be involved in the coronavirus disease 2019 (COVID-19) (Kouhpayeh et al., 2021), based on the facts that oxidative stress plays a major role in the pathogenesis, progression, and severity of COVID-19 (Beltrán-García et al., 2020; Cecchini and Cecchini, 2020; Delgado-Roche and Mesta, 2020) and that infection of the severe acute respiratory syndrome coronavirus-2 (SARS-CoV-2) is known to induce apoptosis and necroptosis (Donia and Bokhari, 2021; Li et al., 2020). TRPM7 was demonstrated to be somehow associated with causes of fibrotic diseases (Du et al., 2010; Fang et al., 2013; Yu M. et al., 2013), cancer malignancy (Yee, 2017), and multiple sclerosis (Kamermans et al., 2019). Thus, further studies are warranted to clarify how TRPM2 and TRPM7 are molecularly and causatively involved in the etiology of these diseases. In any case, TRPM2 and TRPM7 are expected to be attractive targets for future treatments and drug developments.
